# Data-driven optimization of siderophore fermentation by *Bacillus altitudinis* AS19 and investigation of AS19’s antifungal properties against *Candida albicans*

**DOI:** 10.1128/aem.00815-26

**Published:** 2026-08-04

**Authors:** Chenjian Li, Zexin Gao, Honghai Ke, Huake Jia, Hongxia Xiang, Xi Cui, Tao Li, Yue Zhang, Wenping Ding, Hongmei Liu

**Affiliations:** 1 Engineering Research Center of Medical Biotechnology, School of Biology and Engineering, Guizhou Medical University 74628, Guiyang, China; 2 School of Basic Medicine Science, Guizhou Medical University 74628, Guiyang, China; 3 Experimental Animal Center of Guizhou Medical University 74628, Guiyang, China; Chalmers tekniska hogskola AB, Gothenburg, Sweden

**Keywords:** *Candida albicans*, siderophore, fermentation process optimization, virtual screening, antimicrobial activity

## Abstract

*Candida albicans* is a common opportunistic pathogenic fungus capable of causing severe infections when host immunity is compromised or when the normal microbial flora is disrupted. The widespread use of antifungal agents has led to the increasing emergence of drug resistance, underscoring the urgent need for novel antifungal strategies. *Bacillus altitudinis* AS19 has been found to produce a substance with specific antimicrobial activity against *C. albicans* under iron-deficient conditions. In this study, the fermentation medium and culture conditions for siderophore production by AS19 were optimized using response surface methodology (RSM) and an artificial neural network-genetic algorithm (ANN-GA) model. The ANN-GA model demonstrated superior optimization performance compared with RSM. The optimal conditions were pH 6.363, sucrose 29.730 g/L, and KH_2_PO_4_ 0.185 g/L, under which the yield increased more than twofold compared with the pre-optimization level of 26.53%. To further investigate the antimicrobial mechanism, molecular docking and dynamics simulations were employed to virtually screen siderophores with anti-*C*. *albicans* activity and identify their potential targets. Among the candidates, Nocardamin Glucuronide was prioritized as the top hit, and the presence of desferrioxamine-type siderophores produced by AS19 was subsequently confirmed by LC-MS, validating the *in silico* predictions. In addition, *in vitro* assays were performed to determine the minimum inhibitory concentration, minimum fungicidal concentration, and time-kill kinetics of the crude siderophore extract. The time-kill curve clearly demonstrated its potent antimicrobial activity against *C. albicans*. Overall, this study provides both a theoretical basis and experimental evidence for the development of novel siderophore-based antimicrobial agents targeting *C. albicans*.

**IMPORTANCE:**

*Candida albicans* is a common opportunistic fungal pathogen responsible for severe infections in immunocompromised individuals, while increasing antifungal resistance has reduced the effectiveness of current treatments. In this study, a natural compound with specific anti-*C*. *albicans* activity produced by *Bacillus altitudinis* AS19 under iron-deficient conditions was investigated. Artificial intelligence-based models were used to optimize fermentation conditions, significantly improving production efficiency. Molecular simulations were performed to explore the potential antimicrobial mechanism, and *in vitro* assays confirmed its antifungal activity. These results provide a basis for developing novel antifungal agents targeting *C. albicans*.

## INTRODUCTION

*Candida albicans* is a common opportunistic fungal pathogen that frequently colonizes human mucosal surfaces, such as the oral cavity and gastrointestinal tract (1). It can cause infections when host immunity is compromised or microbial homeostasis is disrupted. The extensive use of antifungal agents, particularly azoles, has led to the increasing emergence of drug-resistant *C. albicans* strains (2). Iron is essential for the growth and pathogenicity of *C. albicans*, playing key roles in energy metabolism, DNA synthesis, and hyphal development (3). To acquire iron under iron-limited conditions, microorganisms secrete siderophores—high-affinity iron-chelating compounds that facilitate efficient iron uptake (4). Some microorganisms can also utilize siderophore-iron complexes produced by competitors. In response, certain microbes produce sideromycins, which are conjugates of modified siderophores covalently linked to antimicrobial moieties (5). These compounds act as “Trojan horses,” entering target cells via siderophore uptake systems and subsequently releasing antimicrobial components that lead to cell death (6, 7).

Preliminary studies showed that *Bacillus altitudinis* AS19 produces hydroxamate-type siderophores in SA iron-deficient medium, and its culture supernatant exhibits specific inhibitory activity against *C. albicans*. In contrast, neither siderophore production nor antimicrobial activity is observed in LB broth medium (8), suggesting that the strain may produce sideromycins. The naturally low yield of siderophores limits further investigation, making it essential to optimize fermentation media and culture conditions to enhance their production.

Response surface methodology (RSM) is an efficient optimization tool integrating experimental design, mathematical modeling, and statistical analysis and is widely used in microbial cultivation. Compared with single-factor and orthogonal experiments, RSM can determine optimal fermentation conditions with fewer experiments while improving optimization accuracy and reliability (9). Artificial neural networks (ANNs) are data-driven computational models capable of capturing complex nonlinear relationships; however, they are often regarded as “black-box” models with limited interpretability and potential overfitting issues (10). Genetic algorithm (GA), an evolutionary global optimization method, can efficiently search for optimal solutions in complex parameter spaces without relying on gradient information (11, 12). Therefore, integrating ANN with GA can effectively enhance optimization efficiency and predictive performance in complex nonlinear systems (13).

Although effective in many cases, RSM relies on a fixed model structure (typically a quadratic polynomial), which limits its ability to accurately describe the highly nonlinear, multivariable coupled response surfaces typical of microbial fermentation and may fail to identify the global optimum. More broadly, microbial fermentation is characterized by strong nonlinearity, multivariable coupling, and limited mechanistic prior knowledge. While machine learning models such as support vector machine and random forest can serve as predictors, they often require large data sets or lack compatible interfaces with optimization algorithms (14); metaheuristics like particle swarm optimization and ant colony optimization, in turn, frequently suffer from premature convergence and poor adaptability to continuous spaces (15). To address these challenges, an integrated RSM-ANN-GA strategy was adopted: RSM provided an initial experimental framework, ANN captured the complex nonlinear relationships to improve predictive accuracy without requiring mechanistic priors, and GA performed global optimization on the ANN-derived response surface by exploiting its parallel, gradient-free search capability (16–18). This complementary division of labor, ANN for high-fidelity modeling and GA for robust optimization, makes the hybrid approach particularly suitable for high-dimensional, strongly nonlinear fermentation optimization under limited prior knowledge. Accordingly, a machine learning-based model was developed in this study to optimize siderophore fermentation.

Molecular docking is a computational approach used to predict ligand-receptor binding modes and affinities, enabling rapid preliminary screening of candidate compounds and reducing experimental time and cost (19). However, as a static method, it cannot capture the dynamic nature of molecular interactions. Molecular dynamics (MD) simulations can model atomic-scale motions in a solvated environment, revealing the stability and dynamic behavior of complexes over nanosecond to microsecond timescales and are therefore commonly used to validate docking results (20, 21). In this study, key proteins associated with iron acquisition, growth, and pathogenicity in *C. albicans* were selected as docking targets. The high-affinity iron permease Ftr1 forms a complex with the multicopper oxidase Fet3 to mediate transmembrane Fe^2+^ transport (22). The siderophore transporter Sit1 specifically recognizes and imports siderophore-Fe^3+^ complexes (23). Aspartic protease Sap2, a major virulence factor, degrades various host proteins (24). Sterol 14-α-demethylase (CYP51), an essential enzyme in ergosterol biosynthesis, is critical for maintaining fungal cell membrane integrity (25). These four proteins were therefore selected as targets for molecular docking.

In this study, AS19 was used to optimize siderophore fermentation. A high-precision model integrating RSM with an artificial neural network-genetic algorithm (ANN-GA) was established to optimize culture medium components and fermentation conditions. Whole-genome analysis was performed to predict siderophore biosynthetic gene clusters (BGCs), and potential siderophore candidates were selected based on structural similarity to known compounds. These candidates were further evaluated through molecular docking and molecular dynamics simulations against four key *C. albicans* proteins to identify molecules with potential antifungal activity. In addition, *in vitro* assays were conducted to assess the effects of the selected siderophores on the growth of *C. albicans*. This study provides a theoretical and experimental basis for the development of novel siderophore-based antifungal agents.

## MATERIALS AND METHODS

### Materials

*Bacillus altitudinis* AS19, utilized in this study, was isolated and purified from rhizosphere soil samples of *Paris polyphylla* collected from Yanla Township, Xixiu District, Anshun City, Guizhou Province (105.968°E, 26.041°N). The strain is currently preserved at the School of Biology and Engineering, Guizhou Medical University. *Candida albicans* ATCC 10231 was used to evaluate the antifungal activity.

All chemicals and reagents, including anhydrous piperazine, 5-sulfosalicylic acid, Chrome Azurol S (CAS), iron(III) chloride hexahydrate, cetyltrimethylammonium bromide, amino acids such as asparagine, lysine, tryptophan, phenylalanine, methionine, threonine, isoleucine, leucine, valine, and histidine, carbohydrates such as glucose, fructose, xylose, xylan, lactose, and sucrose, polyols including glycerol, sorbitol, and mannitol, cellulose, inorganic salts including NaCl, CaCl_2_, MgSO_4_, MnCl_2_, BaSO_4_, ZnSO_4_, CuSO_4_, KCl, K_2_HPO_4_, KH_2_PO_4_, concentrated hydrochloric acid, sodium hydroxide, and biochemical reagents such as yeast extract, tryptone, soy peptone, and casein, were purchased from Beijing Solarbio Science & Technology Co., Ltd. The DNA extraction kit was obtained from Tiangen Biotech (Beijing) Co., Ltd.

### Strain culture

LB medium consisted of yeast extract 5 g/L, tryptone 10 g/L, NaCl 10 g/L, pH 7.0 (for solid medium, 1.5% agar was added). SA medium consisted of asparagine 2 g/L, sucrose 20 g/L, K_2_HPO_4_ 1 g/L, MgSO_4_·7H_2_O 1 g/L, pH 7.0 (for solid medium, 1.5% agar was added). Casein-based medium consisted of casamino acids 5 g/L, glucose 1 g/L, yeast extract 1 g/L, soy peptone 1 g/L, KH_2_PO_4_ 0.5 g/L, K_2_HPO_4_ 1 g/L, MgSO_4_ 0.1 g/L, MnCl_2_ 0.1 g/L, NaCl 0.5 g/L. Basal medium consisted of glucose 20 g/L, asparagine 2 g/L, NaCl 0.15 g/L, K_2_HPO_4_ 0.15 g/L, pH 7.0.

#### Preparation of CAS assay solution

Stock solutions were stored at 4°C. For Chrome Azurol S solution, 0.121 g was dissolved and adjusted to a final volume of 100 mL with deionized water. For Fe³^+^ solution, 0.270 g of FeCl₃·6H₂O was dissolved and adjusted to a final volume of 1 L with deionized water. For 5-sulfosalicylic acid solution, 5 g was dissolved and adjusted to a final volume of 100 mL with deionized water. For piperazine solution, 4.307 g of anhydrous piperazine was dissolved in 30 mL of deionized water, and the pH was adjusted to 5.6. For working solution preparation, 0.0219 g of cetyltrimethylammonium bromide was dissolved in 50 mL of deionized water. Under constant stirring, the following solutions were added sequentially: 7.5 mL of Chrome Azurol S stock solution, 4.5 mL of Fe³^+^ stock solution, 4.5 mL of piperazine stock solution, and 2 mL of 5-sulfosalicylic acid stock solution. The final volume was then adjusted to 100 mL with deionized water. The working solution was stored protected from light.

The strain AS19 preserved at −80°C was inoculated at 0.1% (vol/vol) into 50 mL of casein-based medium and cultured in a constant-temperature shaker at 37°C and 200 rpm for 36 h for activation. Subsequently, the bacterial culture was transferred at 1% (vol/vol) inoculum into 100 mL of liquid LB medium and incubated under the same conditions until reaching the logarithmic growth phase (OD_600_ = 0.6) to prepare the seed culture. Finally, the seed culture was added at 5% (vol/vol) inoculum into 50 mL of media with different compositions and cultivated at 37°C and 200 rpm for 48 h. Siderophore production was quantitatively analyzed using the CAS liquid assay. In all shake-flask experiments, 250 mL conical flasks were used.

### Quantitative detection of siderophores

Siderophore production was quantified using the CAS assay to determine relative siderophore content (26, 27). Bacterial suspension of AS19 cultured in SA medium was centrifuged (12,000 rpm, 4°C, 20 min) to obtain cell-free supernatant. The supernatant was then mixed with an equal volume of CAS assay solution in a 96-well plate. After thorough mixing, the reaction was allowed to proceed at room temperature in the dark for 0.5–1 h. Absorbance at 630 nm was measured using a microplate reader. Siderophores induce a color change in the CAS medium, resulting in a decrease in the measured OD_630_ value. Siderophore unit (SU) was calculated as follows:


$$SU=\frac{Ar-As}{Ar}\times 100\%$$

where SU is the relative siderophore content (in siderophore units), Ar is absorbance at 630 nm of the blank (sterile SA medium), and As is absorbance at 630 nm of the sample (cell-free culture supernatant).

### Whole-genome characterization of AS19

#### DNA extraction and whole-genome sequencing analysis of AS19

Genomic DNA of strain AS19 was extracted using a bacterial genomic DNA extraction kit according to the manufacturer’s instructions (Tiangen Biotech [Beijing] Co., Ltd). The extracted DNA was subsequently sent to Sangon Biotech (Shanghai) Co., Ltd. for whole-genome sequencing. Assembly and annotation of the genomic data were subsequently performed.

#### Prediction of siderophore biosynthesis gene clusters in AS19

The potential secondary metabolites and their corresponding biosynthesis gene clusters were predicted using the online bacterial secondary metabolite analysis platform antiSMASH 8.0.4 (28) (thttps://antismash.secondarymetabolites.org/#!/start). The annotated GenBank file was analyzed with antiSMASH 8.0.4 (KnownClusterBlast, ClusterBlast, SubClusterBlast, MIBiG cluster comparison) to predict BGCs and retrieve annotations from the HTML report.

#### Single-factor experiments

Using the basal medium as the initial composition, the effects of 10 different carbon sources, nitrogen sources, and inorganic salts on siderophore production by strain AS19 were systematically evaluated via the single-factor method. Optimal carbon, nitrogen, and inorganic salt sources were identified, followed by assessment of their concentrations (carbon: 10, 15, 20, 25, and 30 g/L; nitrogen: 2, 4, 6, 8, and 10 g/L; inorganic salts: 0.15, 0.20, 0.25, 0.30, and 0.35 g/L) on siderophore yield. Additionally, the influence of pH (6.0, 6.5, 7.0, 7.5, and 8.0), temperature (27, 29, 31, 33, 35, 37, and 39°C), and agitation speed (160, 170, 180, 190, and 200 rpm) on siderophore production was investigated. All experiments were performed in shake flasks, and siderophore relative content (SU) was used to quantify the effect of each factor.

### Response surface optimization experiment

#### Plackett-Burman experimental design

The Plackett-Burman design was constructed using Design-Expert software (29, 30). Based on the results of the single-factor experiments, 11 factors were selected for investigation: pH (A), temperature (B), agitation speed (C), sucrose (D), asparagine (E), ZnSO_4_ (F), CaCl_2_ (G), MgSO_4_ (H), NaCl (J), K_2_HPO_4_ (K), and KH_2_PO_4_ (L) (Table A1). The relative siderophore content (SU) was used as the response value to evaluate the influence of each factor on siderophore production by strain AS19 and to identify the key fermentation parameters.

#### Box-Behnken design and validation

The Box-Behnken design was established using Design-Expert software (9). Based on the results of the Plackett-Burman experiment, three significant factors—pH (A), sucrose (B), and KH_2_PO_4_ (C)—were selected for a three-factor, three-level experimental design (Table A2). The relative siderophore content (SU) was used as the response value to optimize the fermentation process. The predicted optimal fermentation conditions and the corresponding siderophore yield were subsequently validated through shake-flask fermentation cultures.

### Optimization based on artificial neural network and genetic algorithm

#### Artificial neural network model training

The Box-Behnken experimental design and corresponding results were used as the training data set. An artificial neural network (ANN) model was developed using the Neural Fitting Toolbox in MATLAB to predict siderophore fermentation yield (11, 16). The network architecture consisted of an input layer with three nodes (corresponding to the three input factors), a hidden layer with 10 neurons, and an output layer with one node for yield prediction. The Levenberg-Marquardt backpropagation algorithm, known for its excellent convergence, was selected as the training function. A sigmoid activation function was employed to introduce nonlinearity and model the complex relationship between the input factors and the output response. The data set was randomly divided into three subsets: training set (70%), validation set (15%), and test set (15%). The ANN was iteratively trained, and its performance was evaluated accordingly.

#### Optimization using genetic algorithm

The trained weights and parameters of the neural network were encoded as individuals to construct the initial population in the genetic algorithm (GA). The ga function in MATLAB was then invoked, with the fitness function transformed to align with the GA’s maximization framework. The performance of the model on the validation set was used as the fitness criterion. Iterative evolutionary optimization was carried out, ultimately yielding an improved set of network weights (13). Throughout the optimization process, the global best fitness value was tracked to monitor convergence. The best individual (i.e., the optimal weight configuration) was output, and the corresponding optimal input values, along with their predicted yields, were recorded.

### Comparison of optimization results

Shake-flask fermentation was used to validate the optimal outcomes predicted by response surface methodology and genetic algorithm optimization under the optimized medium and conditions. Comparative analysis of the two approaches was performed, and the predictive accuracy of each method was evaluated based on the deviation between predicted and experimental siderophore yields (determined as the relative siderophore content [SU]). This process facilitated mutual verification and further refinement of siderophore production.

### Molecular docking and molecular dynamics simulation

#### Preparation of docking ligands and target proteins

Based on the prediction and alignment of the siderophore biosynthesis gene clusters in AS19, analogous siderophore gene clusters and their corresponding chemical structures were identified. Further comparative analysis was conducted to screen for potential bioactive compounds. The three-dimensional structures of these candidates were constructed using ChemDraw software and energy-minimized to meet the requirements for molecular simulation. Each molecule was then processed in AutoDock Tools, including the addition of Gasteiger charges, definition of rotatable bonds, and addition of hydrogen atoms.

Four proteins were selected as molecular docking targets: the high-affinity iron reductase (Ftr1), aspartic protease (Sap2), siderophore transporter Sit1, and sterol 14-α-demethylase (CYP51). Experimentally resolved crystal structures of the target proteins were retrieved from the RCSB PDB database. For targets without available crystal structures, homology modeling was performed. All protein structures were uniformly preprocessed using AutoDock Tools, including hydrogen addition, charge assignment, and removal of water molecules, metal ions, and non-essential ligands.

#### Molecular docking

Molecular docking was performed using AutoDock Vina, in which 20 candidate ligands were cross-docked against the four target proteins, enabling comprehensive simulation of multiple binding combinations (19). For each docking run, a search space (grid box) was centered on the active site of the corresponding protein and sized to sufficiently cover the primary binding region. Each docking simulation generated nine possible binding poses, and the pose with the lowest binding free energy was selected as the final result for scoring. Following the docking procedure, the optimal binding mode was visualized using PyMOL. Key interactions—such as hydrogen bonds, hydrophobic contacts, and π-π stacking—were analyzed to preliminarily infer the potential molecular mechanism of action.

#### Molecular dynamics simulation

For the high-affinity complexes identified through screening, MD simulations were further conducted to validate their stability under physiological conditions (20, 21). The top 3–5 complex systems were selected and subjected to 100 ns of explicit solvent simulation using GROMACS. The AMBER99SB-ILDN force field was applied for the protein, while the ligand force field parameters were generated using the GAFF framework via ACPYPE or CGenFF tools. The TIP3P water model was employed, and Na^+^/Cl^−^ ions were added to achieve a physiological ion concentration of 0.15 M. The simulation protocol comprised energy minimization, followed by equilibration in the NVT and NPT ensembles (100 ps each), and a final production run. Trajectory analysis focused on several key metrics: backbone root-mean-square deviation (RMSD) for assessing overall structural stability, residue root-mean-square fluctuation (RMSF) for identifying flexible regions, as well as the frequency and duration of hydrogen bond formation. Finally, the gmx_MMPBSA tool was used to extract 100 frames from the trajectory for MM/PBSA binding free energy calculations. By integrating gas-phase energy and solvation effects, a more realistic estimation of binding affinity was obtained, thereby comprehensively evaluating the dynamic behavior and binding strength of the candidate molecules in complex with the target proteins.

### Crude extraction and mass spectrometry analysis of siderophores

Large-scale fermentation of AS19 was carried out using the optimized medium and conditions, affording 2 L of fermentation broth for subsequent extraction. The fermentation broth was centrifuged at 12,000 rpm for 20 min to obtain the cell-free fermentation supernatant. After concentration by rotary evaporation (concentrated approximately 10-fold), the supernatant was dialyzed using a 100 Da dialysis membrane to remove low-molecular-weight compounds. Following dialysis, the sample was freeze-dried to obtain the crude extract of the cell-free fermentation supernatant, designated AS19-1.

Liquid chromatography-mass spectrometry (LC-MS)-based metabolomic profiling was performed on AS19-1 (experimental, 70 mg/mL) and the AS19 fermentation broth (control, stored at −80°C). Each sample (50 µL) was mixed with 20% acetonitrile in methanol containing an internal standard, vortexed (3 min), centrifuged (4°C, 12,000 rpm, 10 min), kept at −20°C for 30 min, and centrifuged again (4°C, 12,000 rpm, 3 min); the supernatant (120 µL) was analyzed. Separation was performed on a Waters HSS T3 column (1.8 µm, 2.1 mm × 100 mm) at 40°C with a 6 min gradient elution (mobile phase A: 0.1% formic acid in water; B: 0.1% formic acid in acetonitrile). Mass spectrometry was carried out on a Q Exactive HF-X in ESI± modes at resolutions of 60,000 (MS1) and 15,000 (MS2). Raw data were converted by ProteoWizard, processed with XCMS, missing values filled by KNN or one-fifth of the minimum, and peak areas normalized by SVR. Metabolites were matched to HMDB and KEGG libraries (score ≥ 0.5, CV < 0.3 in QCs). Differential metabolites were defined by |Log_2_ FC| ≥ 1. Multivariate analyses included principal component analysis (PCA) (R prcomp) and heatmap clustering (ComplexHeatmap), with pathway annotation based on KEGG.

### Investigation of the antifungal activity of AS19-1 against *C. albicans*

The MIC of AS19-1 against *C. albicans* was determined to investigate its antimicrobial activity (31, 32). A single colony of *C. albicans* was obtained by streaking on PDA solid medium. The colony was inoculated into PDA liquid medium and cultured at 28°C with shaking at 200 rpm for 12–16 h. One milliliter of the culture was centrifuged to collect the cells, which were then washed and resuspended in physiological saline, and the cell concentration was adjusted to approximately 1 × 10^8^ CFU/mL. A twofold serial dilution method was used to establish blank, natural growth, solvent control, and AS19-1 experimental groups in a 96-well plate. After incubation at 28°C for 12–16 h, the OD_600_ nm was measured. Appropriate concentrations were selected for gradient dilution and spot plating, and CFUs were counted. The MIC₅₀, MIC₉₀, and minimum fungicidal concentration (MFC) of AS19-1 against *C. albicans* were determined by combining the absorbance values with the CFU counts.

#### Time-kill curve assay

The time-dependent growth curve was used to evaluate the antifungal kinetics of AS19-1 against *C. albicans* (33, 34). Natural growth, solvent control, positive control, and AS19-1 experimental groups (at concentrations of 2× MIC_90_, 1× MIC_90_, and 0.5× MIC_50_) were established. Cultures were incubated at 28°C for 36 h. At 0, 6, 12, 24, and 36 h, OD_600_ nm was measured, and a defined volume of the culture was taken for gradient dilution, spot plating, and CFU enumeration. A growth curve was plotted with time on the *x*-axis and the logarithm of CFU on the *y*-axis.

### Statistical analysis

All experiments were performed in triplicate (three biological replicates). Statistical analyses were conducted using IBM SPSS 27.0, Design-Expert version 13.0.1.0, and MATLAB R2018a, while data visualization was performed using Origin 2025 and GraphPad Prism 8. Molecular docking and molecular dynamics simulations were carried out with ChemDraw 2022, AutoDock Tools, AutoDock Vina 1.2.0, PyMOL, GROMACS 2023, and Discovery Studio 2019.

## RESULTS

### Whole-genome sequencing

#### Gene functional annotation

Functional annotation of the AS19 genome was performed. COG classification (Fig. A1A) revealed genes involved in cell cycle control, cell division, chromosome partitioning, amino acid, nucleotide, and carbohydrate transport and metabolism, translation, transcription, and secondary metabolite biosynthesis. Excluding general prediction or unknown-function categories, the most abundant classes were K (Transcription, 9.72%), E (Amino acid transport and metabolism, 8.81%), and G (Carbohydrate transport and metabolism, 7.71%). GO enrichment analysis (Fig. A1B) showed that biological processes were dominated by cellular process (GO:0009987), metabolic process (GO:0008152), and single-organism process (GO:0044699); cellular components were enriched in organelle (GO:0044464) and cell (GO:0005623); molecular functions were primarily catalytic activity (GO:0003824) and binding (GO:0005488). KEGG pathway enrichment (Fig. A1C) indicated predominant enrichment in metabolism-related pathways, with carbohydrate and amino acid metabolism being the most significant.

COG, KEGG, and GO analyses consistently indicate that the genetic functions of strain AS19 are largely focused on core metabolic activities, with predominant enrichment in carbohydrate and amino acid metabolism pathways. At the biological process level, cellular metabolism and proliferation dominate, while catalytic activity and binding are the primary molecular functions. Cellular component analysis highlights enrichment in cell- and organelle-related structures. These results collectively suggest that AS19 is a metabolically active microorganism with robust capabilities for nutrient utilization, growth, and reproduction.

#### Prediction of siderophore biosynthesis gene clusters in AS19

**Fig 1:**
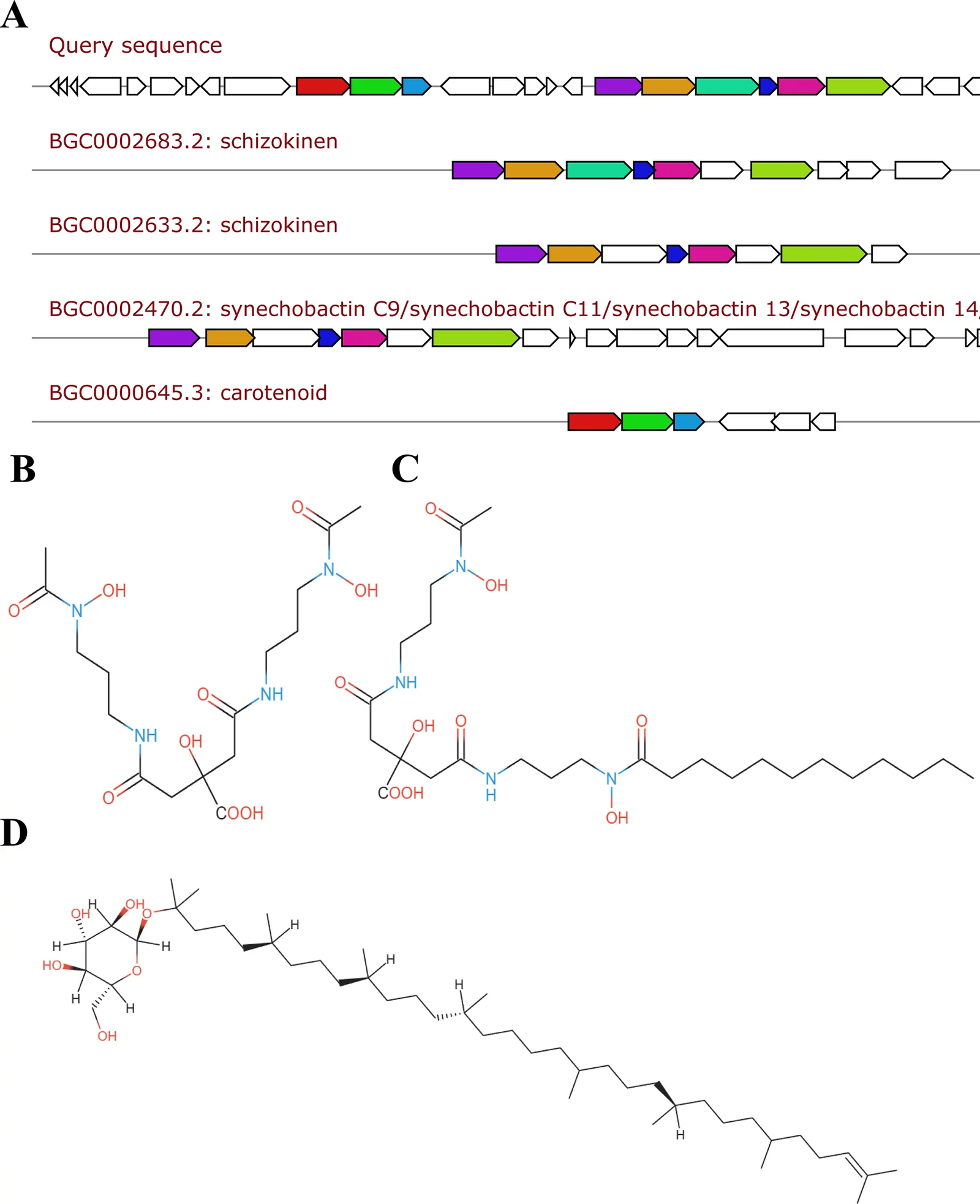
(**A**) Siderophore-similar gene clusters in AS19; (**B**) chemical structure of schizokinen; (**C**) chemical structure of synechobactin A; (**D**) chemical structure of carotenoid.

Secondary metabolite analysis using antiSMASH 8.0.4 (28) identified 11 BGCs, including terpene, T3PKS, RiPP-like, betalactone, NRPS, RRE-containing, and NI-siderophore clusters (Fig. A2). KnownClusterBlast analysis (Fig. 1) revealed that the terpene and NI-siderophore clusters shared the highest similarity with the schizokinen (catecholate-type siderophore) cluster from *Nostoc* sp. PCC 7120 = FACHB-418. MIBiG comparison further indicated that clusters with the highest similarity scores were predominantly siderophore BGCs (Table A1). These results confirm that AS19 harbors a siderophore biosynthetic gene cluster, supporting its potential for siderophore production and guiding subsequent experiments.

### Single-factor experiments

#### Effect of different carbon sources on siderophore production

Carbon sources are essential for microbial growth and metabolism, supplying energy for proliferation and directing metabolic flux, thereby influencing the activation of specific pathways and the biosynthesis of secondary metabolites (35).

**Fig 2:**
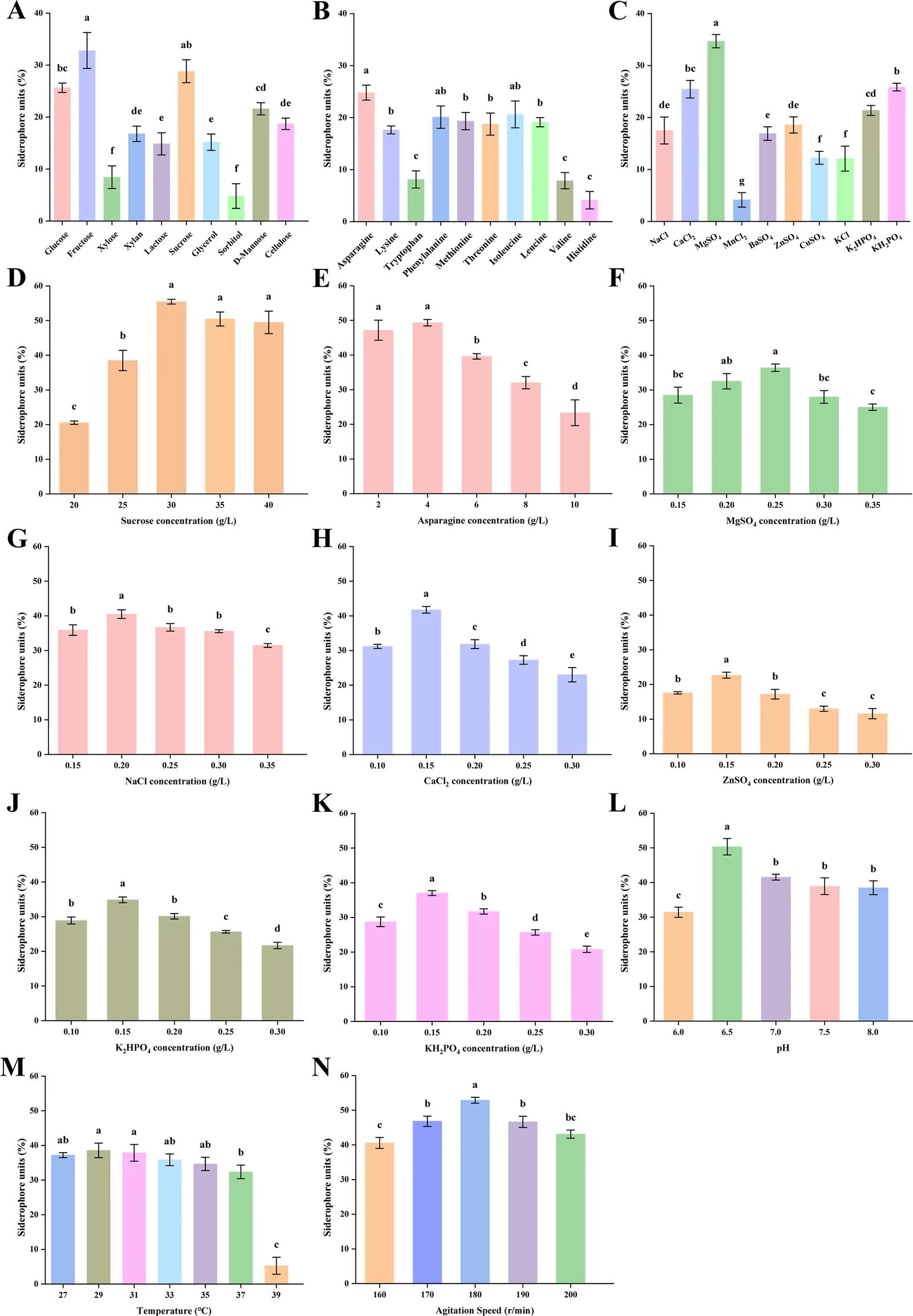
Effects of fermentation medium composition and culture conditions on siderophore production. (**A**) Carbon source; (**B**) nitrogen source; (**C**) inorganic salt; (**D**) sucrose; (**E**) asparagine; (**F**) MgSO_4_; (**G**) NaCl; (**H**) CaCl_2_; (**I**) ZnSO_4_; (**J**) K_2_HPO_4_; (**K**) KH_2_PO_4_; (**L**) pH; (**M**) temperature; (**N**) agitation speed.

To assess the effect of carbon sources on siderophore production, 10 commonly used sugars were compared (Fig. 2A). Fructose, sucrose, and glucose markedly enhanced siderophore yields, with fructose yielding the highest production. Further evaluation of sucrose concentration (Fig. 2D) showed that siderophore yield increased with sucrose up to 30 g/L, then declined at higher concentrations. Optimal carbon levels provide energy and biosynthetic precursors, shorten the lag phase, and promote rapid growth and metabolism, enhancing product synthesis. Excessive carbon, however, raises medium osmotic pressure, induces cellular stress, and decreases pH, inhibiting production (36, 37). AS19 demonstrated good tolerance to high sucrose, and 30 g/L was selected as the optimal concentration for siderophore fermentation.

#### Effect of different nitrogen sources on siderophore production

Nitrogen sources are essential for microbial growth, constituting key cellular macromolecules and regulating metabolic flux by modulating enzyme expression. Appropriate nitrogen sources can enhance anabolic capacity, support cell proliferation, and facilitate the synthesis of certain secondary metabolites (38).

The effects of 10 amino acids on siderophore production were evaluated (Fig. 2B), with yields ranking as follows: asparagine > isoleucine > phenylalanine > methionine > leucine > threonine > lysine > tryptophan > valine > histidine. Asparagine exhibited the strongest promotion of siderophore synthesis and was selected as the optimal nitrogen source. Further investigation of asparagine concentration (Fig. 2E) showed that siderophore yield increased up to 4 g/L, then declined at higher concentrations, indicating that 4 g/L is the optimal concentration for efficient production.

#### Effect of different inorganic salts on siderophore production

Inorganic salts are vital for microbial growth, contributing to metabolism, structural integrity, and physiological homeostasis while supplying essential elements such as nitrogen, phosphorus, and potassium. Appropriate supplementation is therefore crucial for optimizing siderophore production during fermentation.

Ten inorganic salts (NaCl, CaCl_2_, MgSO_4_, MnCl_2_, BaSO_4_, ZnSO_4_, CuSO_4_, KCl, K_2_HPO_4_, and KH_2_PO_4_) were evaluated for their effects on siderophore production (Fig. 2C). The order of influence from highest to lowest was: MgSO_4_ > KH_2_PO_4_ > CaCl_2_ > K_2_HPO_4_ > ZnSO_4_ > NaCl > BaSO_4_ > CuSO_4_ > KCl > MnCl_2_. MgSO_4_ exhibited the strongest promotion, followed by KH_2_PO_4_, CaCl_2_, K_2_HPO_4_, ZnSO_4_, and NaCl. Functionally, KH_2_PO_4_ and K_2_HPO_4_ maintain pH stability (39), NaCl regulates osmotic pressure, CaCl_2_ enhances stress tolerance (40), and ZnSO_4_ serves as a cofactor for enzymatic activity (41). These six salts were selected for supplementation. Optimal concentrations were determined to maximize siderophore yield: MgSO_4_ 0.25 g/L, NaCl 0.20 g/L, CaCl_2_ 0.15 g/L, ZnSO_4_ 0.15 g/L, K_2_HPO_4_ 0.15 g/L, and KH_2_PO_4_ 0.20 g/L (Fig. 2F through K), providing an optimized inorganic salt combination for efficient siderophore synthesis.

#### Effect of different conditions on siderophore production

During fermentation, pH, temperature, and agitation are key factors affecting microbial growth and metabolism (42). pH regulates enzyme activity and nutrient solubility, with AS19 exhibiting maximal siderophore production at pH 6.5; lower pH sharply reduced yield, while weakly alkaline conditions were tolerated (Fig. 2L). Temperature modulates enzymatic reaction rates, with optimal siderophore synthesis at 29°C; higher temperatures induced heat stress and enzyme denaturation, reducing production (Fig. 2M). At 39°C, key enzymes involved in siderophore metabolism were denatured and inactivated, leading to a sharp decline in yield. Agitation influences oxygen transfer and nutrient distribution, with 180 rpm yielding maximum siderophore production; excessive shear or insufficient oxygen limited growth and synthesis (Fig. 2N). Optimization of these physical parameters significantly enhances siderophore production by AS19.

### Response surface optimization experiments

#### Plackett-Burman design experiment

Based on the single-factor experiments, 11 variables were selected for further evaluation: pH (A), temperature (B), agitation speed (C), sucrose (D), asparagine (E), ZnSO_4_ (F), CaCl_2_ (G), MgSO_4_ (H), NaCl (J), K_2_HPO_4_ (K), and KH_2_PO_4_ (L). Each factor was tested at two levels, high (+1) and low (−1) (Table A2). A 12-run Plackett-Burman design was applied to assess their effects on siderophore production (Table 1), and the results were analyzed using Design-Expert software. The Pareto chart of standardized effects is shown in Fig. A3. The analysis indicated that pH, sucrose, ZnSO_4_, and NaCl had negative effects, whereas asparagine, MgSO_4_, CaCl_2_, K_2_HPO_4_, KH_2_PO_4_, temperature, and agitation speed showed positive effects on siderophore yield. ANOVA (Table A3) identified pH, agitation speed, sucrose, ZnSO_4_, and KH_2_PO_4_ as significant factors, with KH_2_PO_4_ exhibiting the strongest influence. Considering its buffering role and significance, KH_2_PO_4_, together with pH and sucrose, was selected for further optimization. Based on these results and the single-factor experiments, the fermentation medium was formulated as follows: KH_2_PO_4_ (to be optimized), sucrose (to be optimized), asparagine (4 g/L), MgSO_4_ (0.25 g/L), CaCl_2_ (0.15 g/L), NaCl (0.15 g/L), K_2_HPO_4_ (0.15 g/L), and ZnSO_4_ (0.1 g/L). The culture conditions were set at pH (to be optimized), 29°C, and 180 rpm.

**TABLE 1:** Plackett-Burman experimental design and results

Run on	Variable											Siderophore units (%)
	A	B	C	D	E	F	G	H	J	K	L	
1	1	−1	−1	−1	1	−1	1	1	−1	1	1	53.06
2	−1	1	1	1	−1	−1	−1	1	−1	1	1	56.76
3	−1	−1	−1	1	−1	1	1	−1	1	1	1	43.66
4	1	1	−1	1	1	1	−1	−1	−1	1	−1	33.48
5	1	−1	1	1	1	−1	−1	−1	1	−1	1	45.86
6	1	−1	1	1	−1	1	1	1	−1	−1	−1	37.65
7	−1	1	1	−1	1	1	1	−1	−1	−1	1	57.04
8	−1	−1	−1	−1	−1	−1	−1	−1	−1	−1	−1	42.46
9	−1	−1	1	−1	1	1	−1	1	1	1	−1	46.30
10	1	1	−1	−1	−1	1	−1	1	1	−1	1	47.26
11	1	1	1	−1	−1	−1	1	−1	1	1	−1	47.18
12	−1	1	−1	1	1	−1	1	1	1	−1	−1	43.34

According to the ANOVA results (Table A3), the model was significant with an F-value of 17.4 (*P* = 0.0075). The coefficient of determination (*R*^2^ = 0.9682) and adjusted *R*^2^ (0.9126) indicate strong agreement between the model and experimental data, explaining over 91% of the observed variability. The coefficient of variation (CV = 4.49%) demonstrates good experimental precision and reproducibility. In addition, the adequate precision value (14.144) exceeded the threshold of 4, confirming a sufficient signal-to-noise ratio for reliable prediction and optimization. The regression equation in coded units is as follows:

Y = 46.17 − 2.09A + 1.34B + 2.29C − 2.71D − 1.94F + 1.22H + 4.44L

#### Box-Behnken design experiment

Based on the Plackett-Burman results, three significant factors—pH (A), sucrose (B), and KH₂PO₄ (C)—were selected for further optimization using a three-factor, three-level response surface design. The factor levels are listed in Table A4, and the experimental design with corresponding results is presented in Table 2. Multiple regression analysis was performed using Design-Expert software to obtain the following quadratic regression equation:

Y = 66.67 − 0.5500A + 0.3700B − 0.0952C + 0.0550AB − 0.5500AC - 0.1150BC − 3.14A^2^ − 2.17B^2^ − 2.70C^2^

**TABLE 2:** Box-Behnken experimental design and results

Run on	Variable			Siderophore units (%)
	A	B	C	
1	–1	1	0	62.05
2	0	0	0	66.59
3	0	0	0	66.91
4	1	0	–1	61.11
5	0	–1	–1	61.04
6	1	1	0	60.87
7	0	1	–1	62.54
8	0	1	1	62.32
9	–1	0	–1	60.92
10	0	–1	1	61.28
11	1	0	1	59.63
12	1	–1	0	60.55
13	–1	0	1	61.64
14	0	0	0	66.56
15	0	0	0	66.12
16	–1	–1	0	61.95
17	0	0	0	67.19

ANOVA results (Table A5) indicated that model terms with *P* < 0.05 were significant. In this model, A, B, AC, A^2^, B^2^, and C^2^ were identified as significant factors. The overall model was highly significant (*P* < 0.0001), while the lack-of-fit was not significant (*P* = 0.3518), confirming good agreement between the model and experimental data. The coefficient of determination (*R*^2^ = 0.9875) and adjusted *R*^2^ (0.9715) further demonstrated the high reliability and predictive ability of the model.

**Fig 3:**
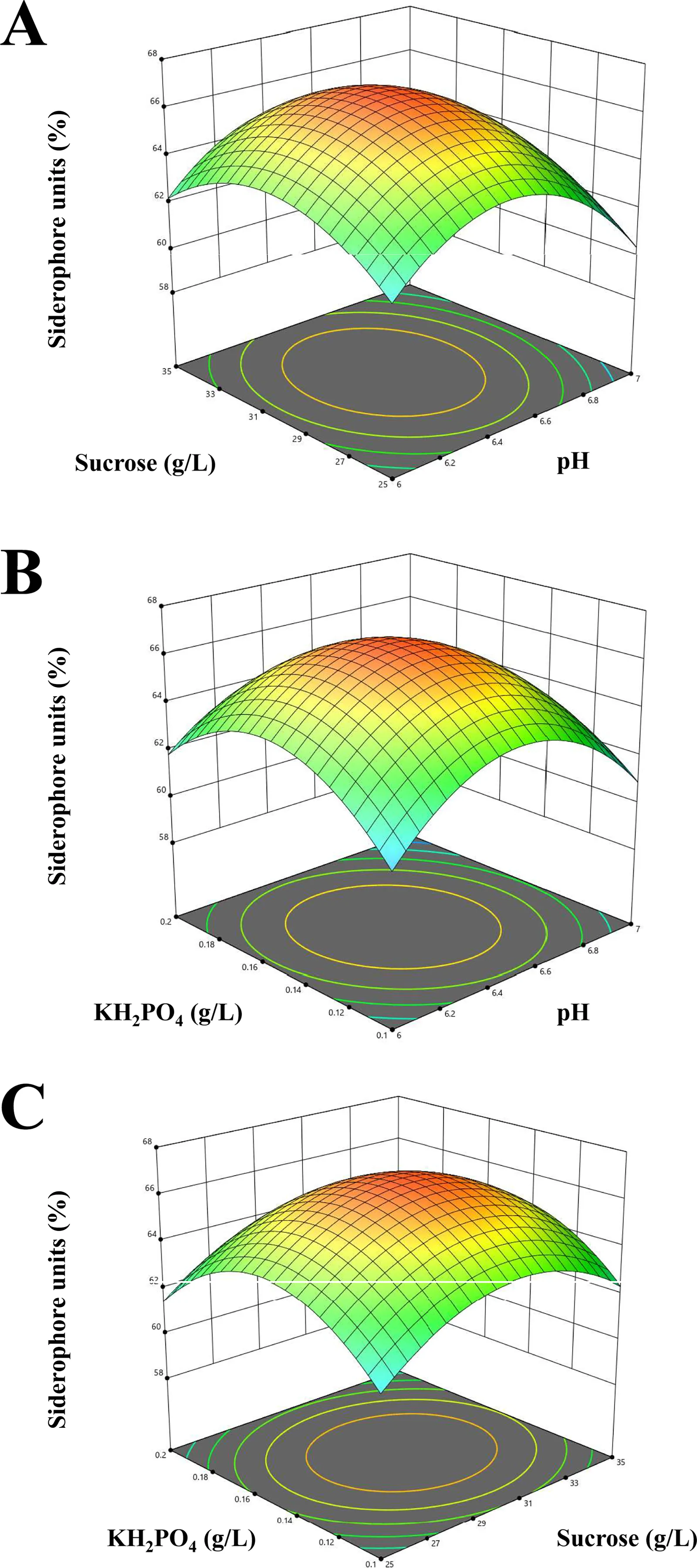
Response surface. (**A**) Interaction surface of pH and sucrose; (**B**) interaction surface of pH and KH₂PO₄; (**C**) interaction surface of sucrose and KH₂PO₄.

The effects of the three variables on siderophore production followed the order: A (pH) > B (sucrose) > C (KH_2_PO_4_). A significant interaction between pH and KH_2_PO_4_ (AC) was observed, suggesting a synergistic effect, likely related to the buffering role of KH_2_PO_4_. Response surface plots illustrating these interactions are shown in Fig. 3. Model optimization using Design-Expert predicted optimal conditions of pH 6.456, sucrose 30.422 g/L, and KH_2_PO_4_ 0.149 g/L, with a predicted siderophore yield of 66.71%.

### Machine learning

#### ANN model development

**Fig 4:**
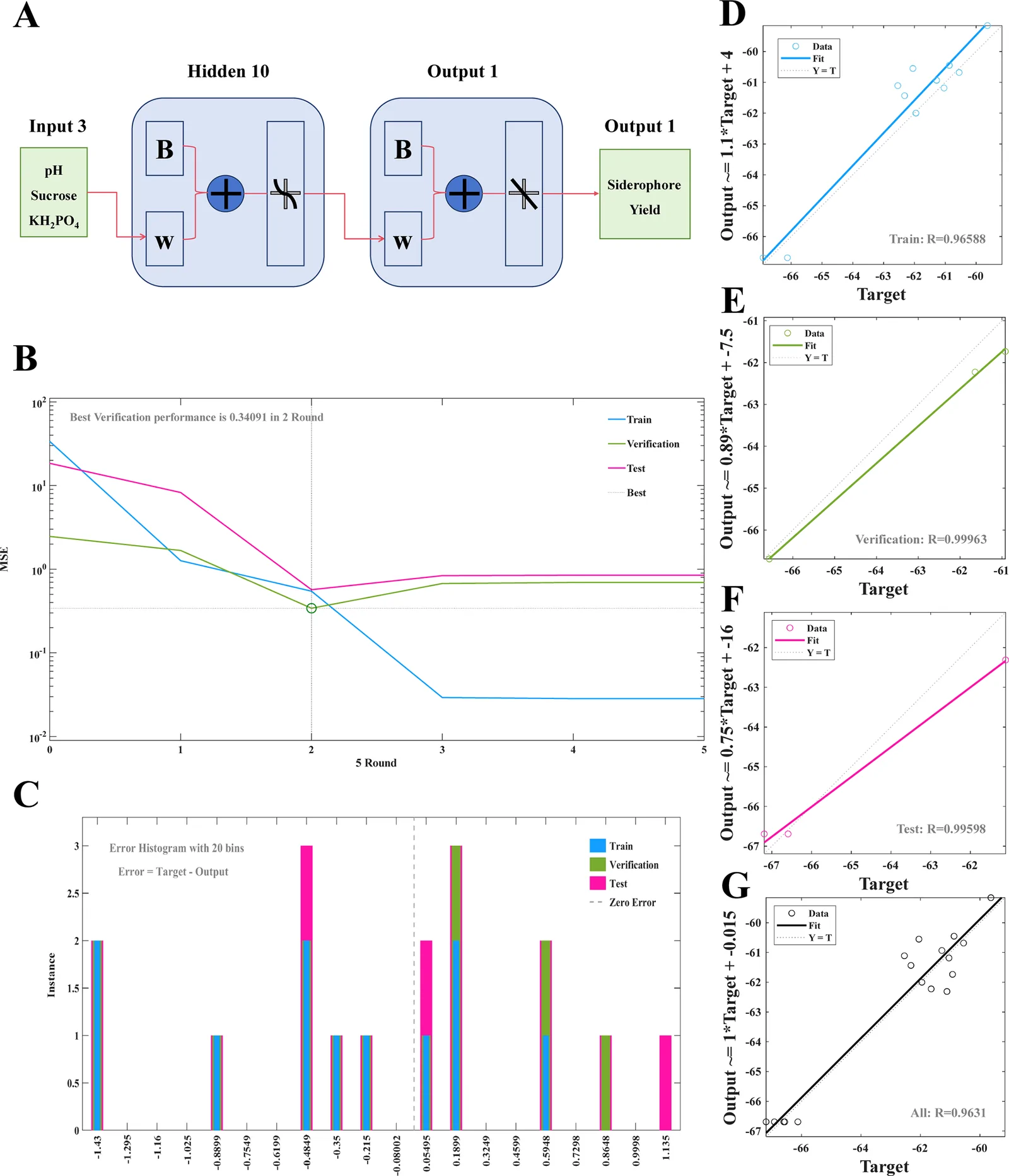
(**A**) Schematic of the neural network architecture; (**B**) MSE variation during the training process; (**C**) error histograms of the training, validation, and test data sets; (**D–G**) regression analysis plots for the training set, test set, validation set, and overall data set.

Based on the response surface results, three key factors—pH, sucrose, and KH_2_PO_4_—were selected as input variables to construct an ANN model for predicting siderophore yield. Seventeen data sets from the Box-Behnken design were used as training samples. The network architecture consisted of three input nodes, 10 hidden neurons, and one output node (Fig. 4A).

The variation of mean squared error (MSE) for the training, validation, and test sets is shown in Fig. 4B. The best validation performance occurred at epoch 2 with an MSE of 0.34091. The rapid decline and subsequent stabilization of MSE during training indicate effective learning and stable model performance without significant overfitting.

Error histograms (Fig. 4C) showed that most prediction errors were concentrated near zero, demonstrating high prediction accuracy. Regression analysis (Fig. 4D through G) yielded correlation coefficients (*R*) of 0.9658 for the training set, 0.9995 for the validation set, 0.9959 for the test set, and 0.9631 for the overall data set, confirming the strong predictive capability of the ANN model.

### GA optimization for the optimal combination

**Fig 5:**
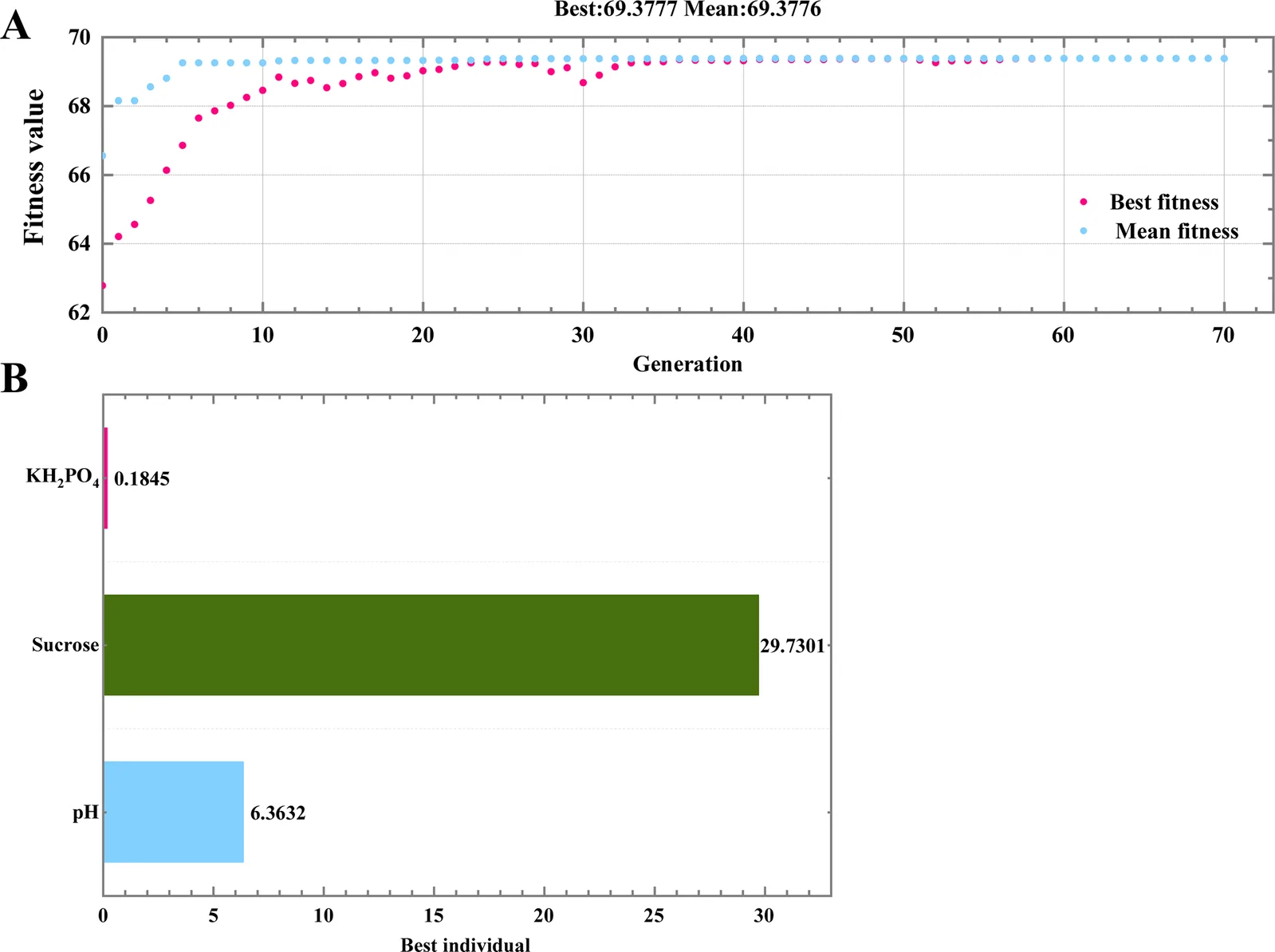
(**A**) Iteration process of the genetic algorithm; (**B**) optimal results predicted by optimization.

Based on the ANN model, a GA was applied to optimize the three significant fermentation factors. As shown in Fig. 5A, the fitness value increased with successive generations and stabilized after 58 iterations, indicating effective convergence, with a maximum fitness value of 69.38%. The optimal solution obtained (Fig. 5B) corresponded to pH 6.363, sucrose 29.730 g/L, and KH_2_PO_4_ 0.185 g/L.

### Comparison of optimization results

As shown in Table 3, the optimal conditions predicted by RSM were pH 6.456, sucrose 30.422 g/L, and KH_2_PO_4_ 0.149 g/L. Under these conditions, three replicate experiments yielded an average siderophore production of 64.43%, with a deviation of ±3.42% from the predicted value (66.71%), confirming the reliability of the RSM model. The ANN-GA model predicted optimal conditions of pH 6.363, sucrose 29.730 g/L, and KH_2_PO_4_ 0.185 g/L. Validation experiments under these conditions produced an average yield of 68.13%, with a deviation of ±1.80% from the predicted value (69.38%), indicating high predictive accuracy.

**TABLE 3:** Comparison of predicted and actual values from RSM and ANN-GA model optimization

Model	Optimal conditions			Siderophore units (%)		
	pH	Sucrose	KH_2_PO_4_	Predicted value	Actual value	Relative error (%)
RSM	6.456	30.422	0.149	66.71	64.43	3.42
ANN-GA	6.363	29.730	0.185	69.38	68.13	1.80

Compared with RSM, the ANN-GA model showed higher predicted and experimental yields and a smaller prediction error, demonstrating superior optimization performance. Accordingly, the final optimal fermentation conditions for siderophore production by AS19 were determined as follows: KH_2_PO_4_ 0.185 g/L, sucrose 29.730 g/L, asparagine 4 g/L, MgSO_4_ 0.25 g/L, CaCl_2_ 0.15 g/L, NaCl 0.15 g/L, K_2_HPO_4_ 0.15 g/L, and ZnSO_4_ 0.1 g/L; culture conditions of pH 6.363, 29°C, and 180 r/min.

### Molecular docking and molecular dynamics simulations

#### Acquisition of ligands and target proteins

Through antiSMASH analysis and comparative screening (Table A1), 10 secondary metabolite biosynthetic gene clusters were identified. Among them, two clusters produced redundant predictions, and one lacked structural information; therefore, seven clusters were retained for downstream compound screening. For predicted siderophore-like products, SMILES structures were extracted and subjected to similarity searches against the NCBI and COCONUT (coconut.naturalproducts.net) databases.

**Fig 6:**
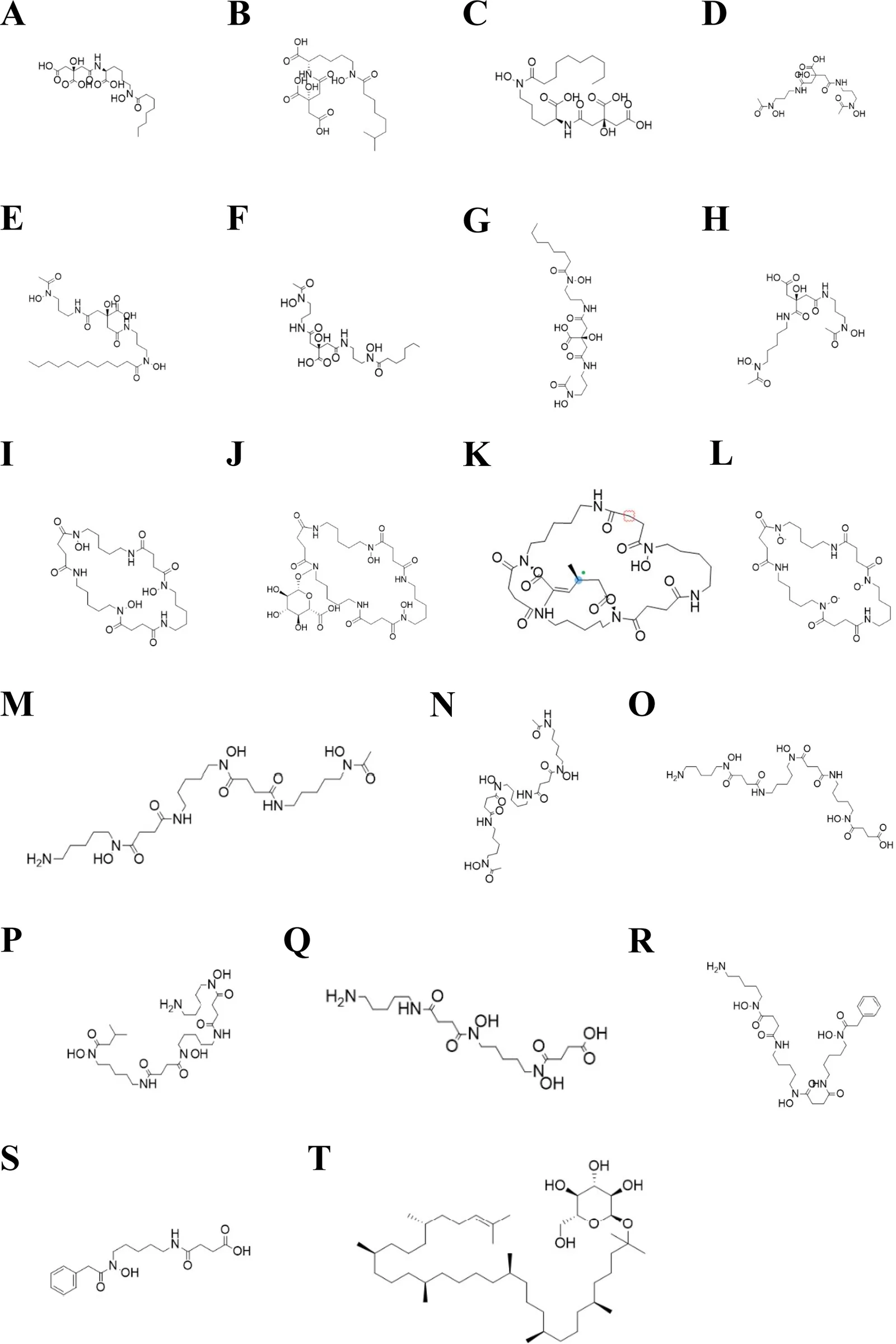
Chemical structures of 20 AS19 siderophore candidates from virtual screening. (**A**) Woodybactin A; (**B**) Woodybactin B; (**C**) Woodybactin D; (**D**) Schizokinen; (**E**) Synechobactin A; (**F**) Synechobactin B; (**G**) Synechobactin C; (**H**) Fradimine B; (**I**) Nocardamine; (**J**) Nocardamin Glucuronide; (**K**) Clavamate B; (**L**) Ferrioxamine E; (**M**) Deferoxamine; (**N**) Desferrioxamine D1; (**O**) Desferrioxamine G; (**P**) Tenacibactin D; (**Q**) Bisucaberin B; (**R**) Legonoxamine A; (**S**) Legonoxamine B; (**T**) Carotenoid.

As shown in Fig. 6, cluster BGC0002469.2 matched Woodybactin A, B, and D in COCONUT. BGC0002683.2 yielded Schizokinen (NCBI) and Synechobactins A–C and Fradimine B (COCONUT). BGC0001572.3 matched Nocardamine, Nocardamin Glucuronide, Clavamate B, and Ferrioxamine E. BGC0000941.3 corresponded to Deferoxamine, Desferrioxamine D1 and G, and Tenacibactin D. BGC0001531.5 matched Bisucaberin B, while BGC0002305.2 yielded Legonoxamines A and B. BGC0000645.3 was annotated as a carotenoid-related cluster. Corresponding small-molecule structures were retrieved for further analysis.

All compounds were drawn using ChemDraw and converted into three-dimensional conformations in Chem3D, followed by energy minimization using the MMFF94 force field to obtain stable low-energy structures. Subsequently, ligands were prepared in AutoDock Tools by adding Gasteiger charges, defining rotatable bonds, adding hydrogens, and assigning rotatable centers, and were saved in PDBQT format for molecular docking (Fig. A4).

**Fig 7:**
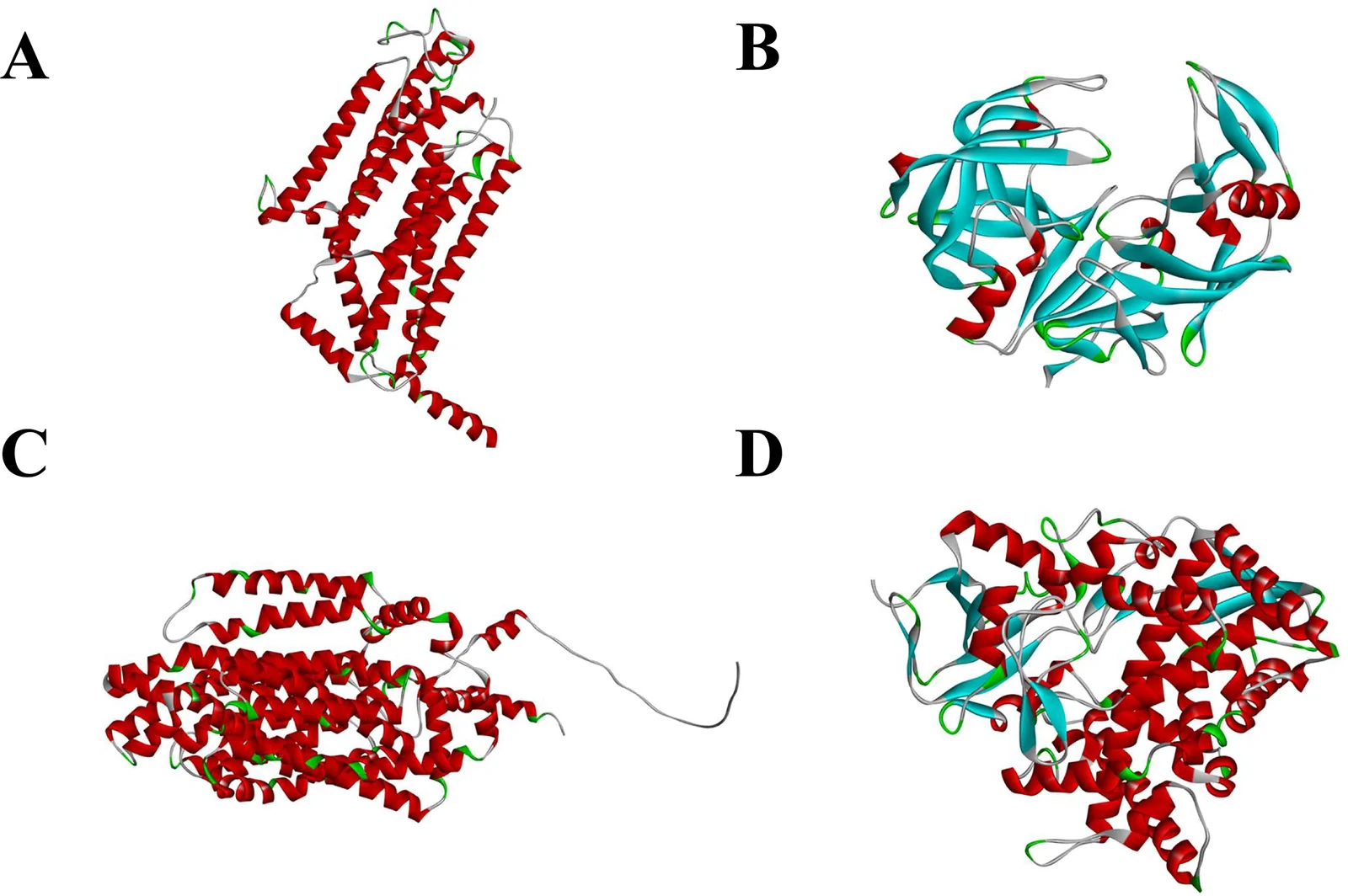
Three-dimensional models of the four target proteins. (**A**) High-affinity iron reductase (Ftr1); (**B**) aspartic protease (Sap2) has an experimentally resolved crystal structure; (**C**) siderophore transporter Sit1; (**D**) sterol 14-α-demethylase (CYP51) has an experimentally resolved crystal structure. The PDB files with IDs 2QZX (resolution 2.50 Å) for Sap2 and 5TZ1 (fluconazole-bound state, resolution 2.00 Å) for CYP51 were downloaded from the RCSB PDB database.

Based on the antifungal activity of the cell-free supernatant from iron-deficient cultures of AS19 against *C. albicans*, four key proteins associated with iron acquisition, growth, and pathogenicity—high-affinity iron reductase (Ftr1), aspartic protease (Sap2), siderophore transporter (Sit1), and sterol 14-α-demethylase (CYP51)-were selected as molecular docking targets. Experimental structures of Sap2 (PDB ID: 2QZX, 2.50 Å) and CYP51 (PDB ID: 5TZ1, fluconazole-bound, 2.00 Å) were obtained from the RCSB PDB database. For Ftr1 and Sit1, homology models were constructed using SWISS-MODEL based on sequences retrieved from the Candida Genome Database. Model evaluation yielded GMQE scores of 0.74 (Ftr1) and 0.85 (Sit1), indicating reliable structural quality. All protein structures were preprocessed in AutoDock Tools by adding hydrogens and charges and removing water molecules, metal ions, and non-essential ligands (Fig. 7).

#### Molecular docking

In this study, 20 ligands were cross-docked against four target proteins, and the binding pose with the lowest binding free energy was selected as the optimal result. Binding energies below −5.0 kcal/mol were considered indicative of potential binding activity, while values below −7.0 kcal/mol were prioritized as strong candidates. Overall, six compounds—Woodybactin A, Ferrioxamine E, Legonoxamine A, Clavamate B, Nocardamin Glucuronide, and Nocardamine—showed strong binding affinity toward all four targets, with most binding energies below −7.0 kcal/mol (Table A6).

**Fig 8:**
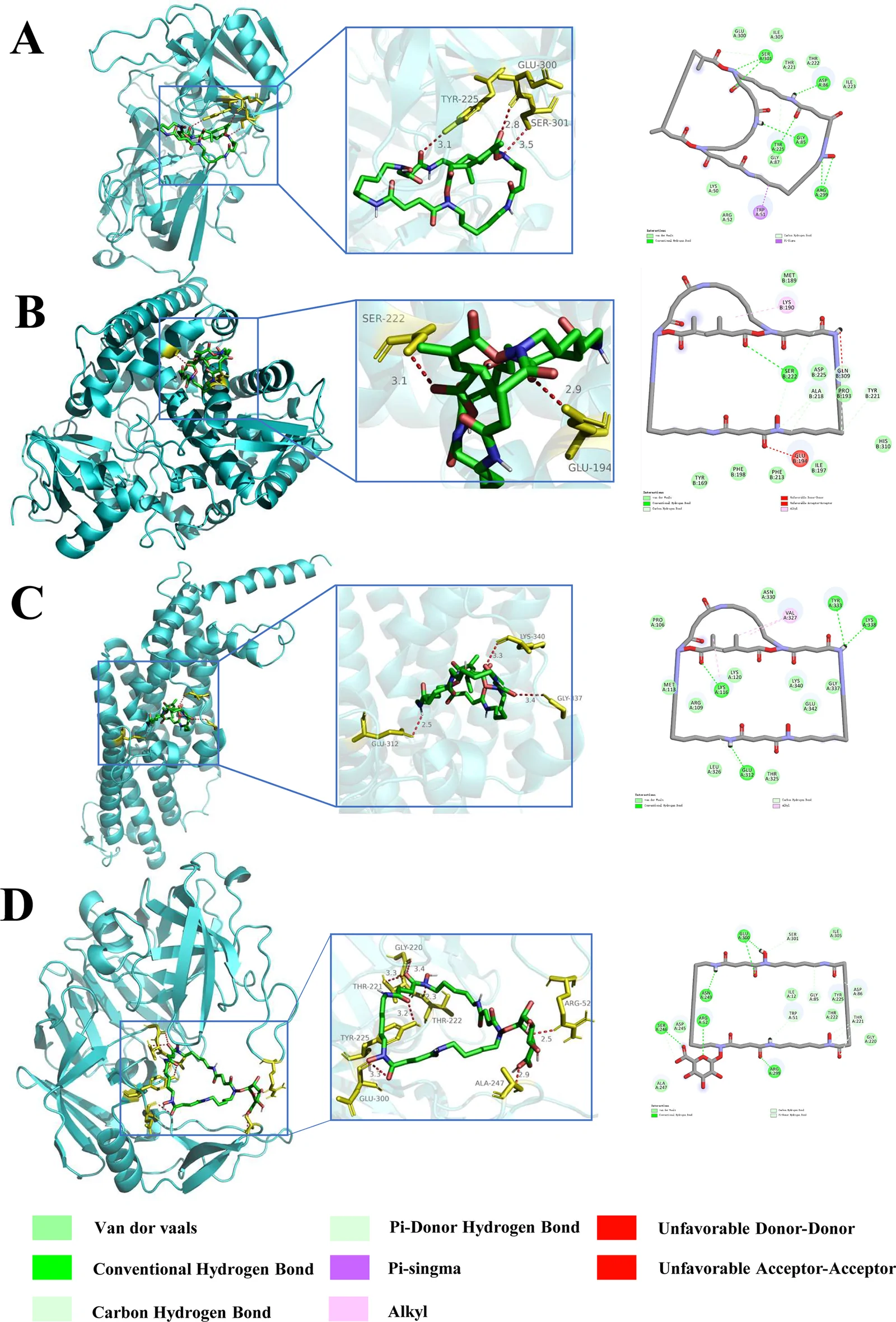
Visualization of the four optimal docking results. (**A**) Clavamate B-2QZX; (**B**) Clavamate B-5TZ1; (**C**) Clavamate B-FTR1; (**D**) Nocardamin Glucuronide-2QZX.

The optimal docking poses of 24 representative complexes were visualized using PyMOL, and protein-ligand interactions were further analyzed using Discovery Studio. As shown in Fig. 8, key residues in Sap2 (2QZX), including TYR-225, THR-222, ILE-223, GLY-220, ARG-299, and ASP-86, mediated hydrogen bonding and stabilizing interactions. In CYP51 (5TZ1), HIS-468, TYR-132, THR-411, GLU-420, and TYR-505 were identified as key binding residues, with HIS-468 directly coordinating the heme iron, suggesting potential disruption of electron transport and sterol biosynthesis (43). In Ftr1, TYR-230, GLU-89, ASP-86, ARG-226, ASN-172, and TRP-52 contributed to ligand recognition via hydrogen bonding and electrostatic interactions. In Sit1, THR-105, SER-388, GLU-468, and TYR-481 were primarily involved in stabilizing ligand binding.

Notably, Clavamate B, Nocardamin Glucuronide, and Nocardamine consistently exhibited strong binding across all targets, with average binding energies below −8.5 kcal/mol, particularly against Sap2 (2QZX) and CYP51 (5TZ1). These results suggest that these compounds may act as broad-spectrum inhibitors targeting iron uptake and sterol biosynthesis pathways, indicating potential antifungal activity.

#### Molecular dynamics simulations

Based on the molecular docking results, the 12 complexes with the highest binding affinities were selected for molecular dynamics simulations. Four representative systems showing optimal performance were identified: Sap2-Nocardamin Glucuronide, Sap2-Nocardamine, CYP51-Nocardamin Glucuronide, and Ftr1-Nocardamine (Fig. A6 to A9).

RMSD analysis demonstrated that all complexes reached stable conformational states during the simulations (Fig. A6). The Sap2-Nocardamin Glucuronide complex stabilized within 2.0–3.0 Å after an initial increase, while Sap2-Nocardamine remained stable within 2.5–3.5 Å. The CYP51-Nocardamin Glucuronide complex exhibited the lowest fluctuations (1.5–2.5 Å), and the Ftr1-Nocardamine system stabilized after ~50 ns. Radius of gyration and solvent-accessible surface area analyses showed only minor fluctuations, indicating that the protein complexes possess a certain degree of conformational flexibility during the dynamic process (Fig. A7). Hydrogen bond analysis revealed stable interaction networks across all complexes (Fig. A8), with multiple hydrogen bonds maintained throughout the simulations. RMSF analysis further indicated that residue flexibility was generally low (<2 Å), particularly at the binding sites, highlighting the high conformational rigidity and binding stability of the complexes (Fig. A9). Overall, these results demonstrate that the four complexes exhibit stable binding conformations and robust intermolecular interactions during the MD simulations.

### Crude extraction and mass spectrometry analysis of siderophores

**Fig 9:**
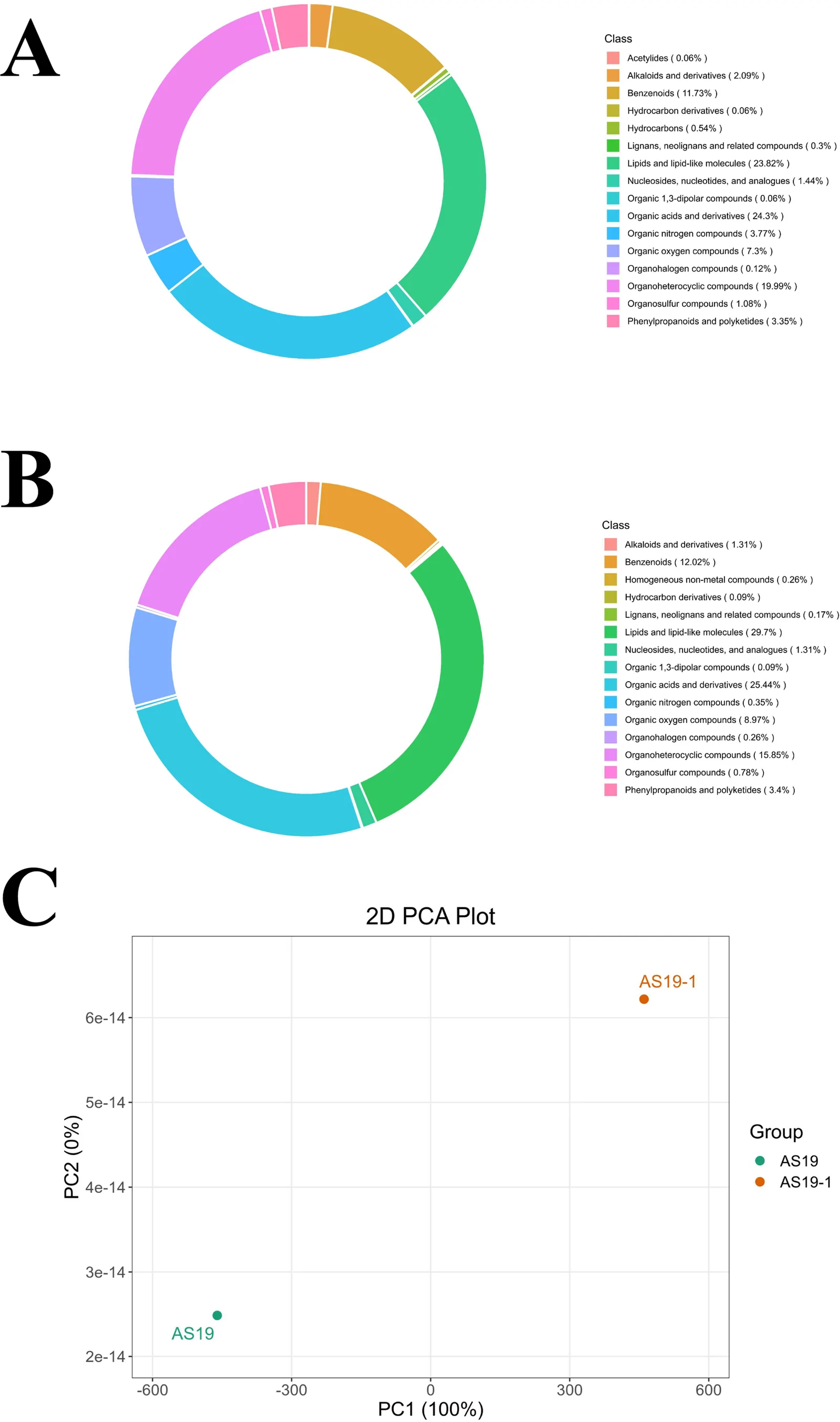
(**A**) Metabolite class composition in positive ion mode (ring chart); (**B**) metabolite class composition in negative ion mode (ring chart); (**C**) PCA plot of the overall samples.

In this study, the crude extract AS19-1 was dissolved in purified water to obtain a 70 mg/mL solution and analyzed by LC-MS to characterize its chemical composition. A total of 15 major compound classes were identified (Fig. 9A and B), including organic acids and derivatives, lipids and lipid-like molecules, organoheterocyclic compounds, benzenoids, alkaloids and derivatives, nucleosides and analogs, organosulfur compounds, and phenylpropanoids and polyketides. Among these, organic acids and derivatives, lipids and lipid-like molecules, organoheterocyclic compounds, benzenoids, and oxygen-containing organic compounds were the predominant constituents in terms of both abundance and diversity. Notably, the siderophore desferrioxamine was identified within the organic acids and derivatives category.

Non-targeted metabolomics analysis was subsequently performed using the AS19 fermentation supernatant as the control and the crude extract AS19-1 as the experimental group. A total of 2,495 metabolic features were detected in both positive and negative ion modes. PCA showed clear separation between the two groups (Fig. 9C), indicating that the purification process significantly altered the metabolite profile. Differential metabolites were screened using orthogonal partial least squares discriminant analysis combined with variable importance in projection values and fold-change analysis (FC ≥ 2 or FC ≤ 0.5), resulting in the identification of 1,247 significantly differential metabolites.

All detected metabolites were shared between the two groups, and no unique compounds were found in the purified sample (Fig. A10), indicating that the extraction procedure introduced no exogenous contaminants and maintained high specificity. Among the significantly upregulated metabolites after purification, desferrioxamine showed prominent enrichment. Semi-quantitative analysis further indicated that the relative content of desferrioxamine increased by 21.96-fold compared with the original supernatant (Table A8), with a similarity score of 0.7232 to the reference compound (CAS: 70-51-9) (44, 45), confirming the effective enrichment of siderophore-type metabolites.

### Investigation of the antifungal activity of AS19-1 against *C. albicans*

**Fig 10:**
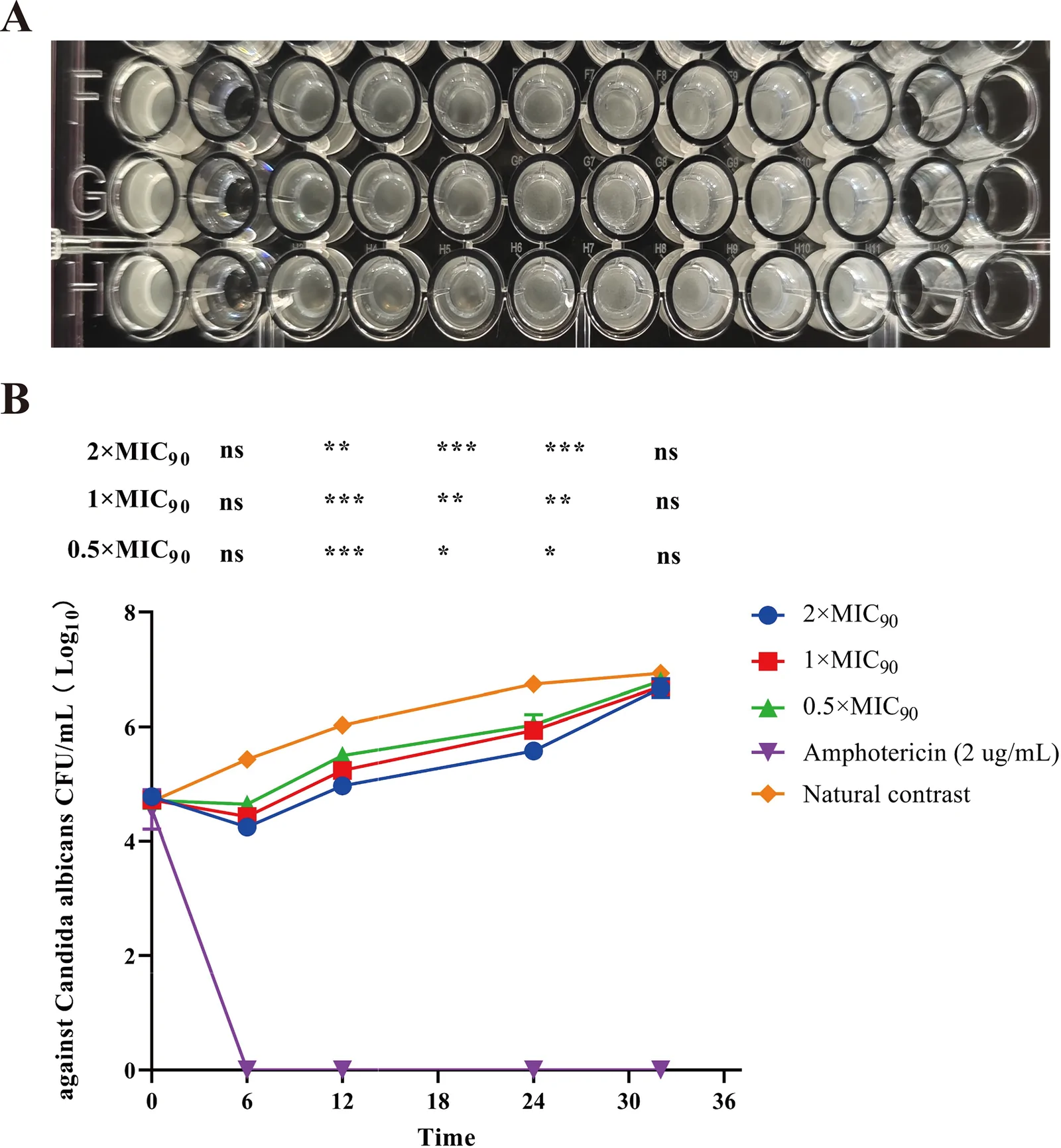
(**A**) MIC of AS19-1; (**B**) time-kill kinetics of AS19-1. Twofold dilutions of AS19-1 (left to right: well 1, natural control; well 2, blank; well 3, 5 mg/mL; then twofold serial dilutions) in a 96-well plate. Statistically significant differences are indicated by * *P* < 0.05, **P < 0.02, and ***P < 0.001.

To evaluate the antifungal activity of AS19-1, the MIC against *C. albicans* was first determined. AS19-1 was prepared as a 20 mg/mL stock solution for testing. As shown in Fig. 10A, the MIC_50_, MIC_90_, and MFC were 312.5 μg/mL, 1.75 mg/mL, and 2.50 mg/mL, respectively.

Based on these results, time-kill curve analysis was performed using MIC₉₀ as the reference concentration to evaluate the antifungal kinetics of AS19-1. As shown in Fig. 10B, AS19-1 at 0.5× MIC_90_, 1× MIC_90_, and 2× MIC_90_ significantly inhibited the growth of *C. albicans*. The strongest inhibitory effect was observed after 6 h of treatment, followed by a gradual decline over time. This trend suggests that AS19-1 primarily exerts a fungistatic effect rather than direct fungicidal activity.

## DISCUSSION

Previous studies demonstrated that this strain produces hydroxamate-type siderophores under iron-deficient conditions, and the corresponding cell-free supernatant exhibits significant antifungal activity against *C. albicans*. In contrast, supernatants from iron-sufficient cultures neither produced detectable siderophores nor showed antifungal activity (8). To evaluate the selectivity of this antimicrobial effect, seven bacterial species—*Klebsiella pneumoniae*, *Pseudomonas aeruginosa*, *Acinetobacter baumannii*, *Staphylococcus aureus*, *Escherichia coli*, *Fusobacterium nucleatum*, and *Porphyromonas gingivalis*—were used as indicator strains (Table A9). The results showed that the supernatant exhibited pronounced inhibitory activity only against *C. albicans*, indicating specific antifungal selectivity.

Sideromycins function as “Trojan horses,” entering target cells through siderophore uptake systems and subsequently releasing antimicrobial moieties that lead to cell death (6, 7). Based on these observations, it is hypothesized that the antimicrobial substance produced by AS19 may act as a natural sideromycin, thereby exerting a distinctive anti-*C. albicans* effect (46).

In this study, by integrating molecular docking and molecular dynamics simulations, the interaction mechanisms between siderophore molecules derived from AS19 and key target proteins of *C. albicans* were systematically investigated. The results indicate that nocardamine-type compounds exhibit high binding affinity and stable interactions with multiple targets, with Nocardamin Glucuronide showing particularly representative interactions with Sap2, CYP51, and Ftr1.

Sap2 is a major virulence factor of *C. albicans*, involved in host mucosal degradation and immune evasion (24). Nocardamin Glucuronide exhibited strong binding affinity (−10.0 kcal/mol) and stable conformational behavior (RMSD 2.0–3.0 Å) with Sap2, suggesting a potential reduction in fungal pathogenicity through inhibition of this virulence factor. CYP51 is a key enzyme in the ergosterol biosynthesis pathway and a classical antifungal target (47). The CYP51-Nocardamin Glucuronide complex showed the highest dynamic stability among the systems (RMSD 1.5–2.5 Å). Notably, its binding site partially overlapped with but differed from that of conventional azole drugs, suggesting a potential non-classical inhibitory mechanism. Additionally, the compound may interfere with enzymatic activity through chelation of the heme iron in the CYP51 active site (48), providing a possible strategy to overcome azole resistance. Ftr1 is a core component of the high-affinity iron uptake system in *C. albicans* and is essential for iron acquisition under iron-limited conditions (22). Although Nocardamine formed fewer hydrogen bonds with Ftr1, the complex maintained favorable overall stability, suggesting that it may block iron transport by occupying the iron-binding site and induce intracellular iron depletion, thereby exerting an iron-starvation antifungal effect.

Overall, Nocardamin Glucuronide exhibited multi-target binding toward Sap2, CYP51, and Ftr1, providing a molecular basis for the anti-*Candida* activity of AS19 extracts. This multi-target interaction pattern is consistent with the synergistic characteristics commonly observed in natural products (49, 50). Structurally, most high-affinity siderophores contain multiple hydroxyl, carboxyl, or hydroxamic acid groups, enabling stable hydrogen bonding through polar interactions, while their hydrophobic backbones embed within protein pockets to enhance binding stability. Tyrosine residues (e.g., TYR-225, TYR-132, TYR-198, and TYR-455) frequently function as key hydrogen bond donors or acceptors, suggesting a conserved role in siderophore-protein interactions. These structure-activity relationships provide valuable insights for the future optimization of siderophore-based antifungal compounds.

Molecular docking and molecular dynamics simulations indicated that two desferrioxamine-class compounds—Nocardamine and its glucuronidated derivative Nocardamin Glucuronide—exhibited the strongest binding affinities toward the three target proteins Sap2, CYP51, and Ftr1. LC-MS analysis further confirmed that the siderophore produced by AS19 fermentation belongs to the desferrioxamine class, consistent with the computational predictions. Dialysis experiments showed that the antimicrobial active component passed through a 500 Da dialysis membrane but was retained by a 100 Da membrane, suggesting a molecular weight between 100 and 500 Da and further suggesting that desferrioxamine may be the active compound in this system (45).

Collectively, the results suggest that the active compound may enter *C. albicans* cells via the Sit1 transporter through a “Trojan horse” strategy and exert a distinctive multi-target mode of action. Specifically, it may interfere with ergosterol biosynthesis through non-classical binding to CYP51, block iron uptake by occupying the Ftr1 site, and simultaneously inhibit the virulence factor Sap2. This coordinated disruption of key pathways associated with fungal survival and pathogenicity may explain the high selectivity of the compound, which showed no activity against the seven tested bacterial strains but exhibited specific antifungal activity toward *C. albicans*.

Time-kill assays further indicated that the compound primarily exerts a fungistatic effect, consistent with a mechanism involving metabolic suppression induced by intracellular iron deprivation. Overall, these findings not only demonstrate the antifungal potential of siderophores derived from strain AS19 but also highlight the feasibility of targeting the Sit1-Ftr1 iron transport axis as a novel gateway for antifungal drug delivery. This study therefore provides a theoretical basis and potential lead structures for the development of siderophore-mediated targeted antifungal therapies.

### Conclusion

In this study, strain AS19 was systematically investigated through fermentation optimization, genomic mining, virtual screening, and experimental validation. Compared with the SA medium and fermentation conditions reported previously, the combined optimization strategy using RSM and an ANN-GA nearly doubled siderophore production. The ANN-GA model demonstrated higher optimization efficiency and predictive accuracy than RSM alone. Key factors affecting siderophore synthesis (pH, sucrose, and KH_2_PO_4_) and their optimal combinations were identified, providing a theoretical basis for fermentation scale-up and medium optimization. Genome mining further predicted gene clusters associated with siderophore biosynthesis and antimicrobial activity. Molecular docking and molecular dynamics simulations were subsequently applied to screen potential antifungal siderophore candidates. LC-MS analysis confirmed that the siderophores produced by AS19 belong to the desferrioxamine class, consistent with the compounds predicted by computational screening.

Overall, this study established an efficient fermentation optimization strategy, identified key factors regulating siderophore production in AS19, and integrated genomics with computational analysis to preliminarily reveal the siderophore type and potential antifungal targets. These findings provide a foundation for future siderophore purification, mechanistic studies, and potential applications in antifungal drug development.

## Data Availability

The raw data are available from the corresponding author upon reasonable request. In this study, the COCONUT accession numbers for the natural products are as follows: CNP0536492.1 (Woodybactin A), CNP0352914.0 (Bisucaberin B), CNP0563786.0 (Ferrioxamine E), CNP0476801.0 (Legonoxamine A), CNP0543593.1 (Woodybactin B), CNP0581079.1 (Woodybactin D), CNP0326188.0 (schizokinen), CNP0461430.0 (Synechobactin A), CNP0428246.0 (Synechobactin B), CNP0431181.0 (Synechobactin C), CNP0576521.1 (Clavamate B), CNP0100564.1 (Nocardamin Glucuronide), CNP0496303.0 (Nocardamine), CNP0336264.0 (Deferoxamine), CNP0081014.0 (Desferrioxamine D1), CNP0343712.0 (Desferrioxamine G), CNP0389765.1 (Fradimine B), CNP0466436.0 (Legonoxamine B), and CNP0168022.0 (Tenacibactin D). Additionally, PubChem CID 11227325 corresponds to carotenoid, and CID 2973 (which also has a COCONUT ID) corresponds to deferoxamine. Regarding the proteins, PDB ID 2QZX corresponds to aspartic protease 2 (Sap2), and 5TZ1 corresponds to sterol 14-α-demethylase (CYP51); CGD IDs CAL0000196424 and CAL0000185056 correspond to Sit1 and Ftr1 proteins, respectively.

## SUPPLEMENTAL MATERIAL

Fig. A12 aem.00815-26-s0001.docx Total ion current (TIC) chromatograms of AS19 and AS19‑1.

Supplemental material aem.00815-26-s0002.docx Fig. A1 to A11; Tables A1 to A9.

ASM does not own the copyrights to Supplemental Material that may be linked to, or accessed through, an article. The authors have granted ASM a non-exclusive, world-wide license to publish the Supplemental Material files. Please contact the corresponding author directly for reuse.
