# Supplementary material for: Data-driven optimization of siderophore fermentation by Bacillus altitudinis AS19 and investigation of AS19’s antifungal properties against Candida albicans
Source: Appl Environ Microbiol. 2026 Aug 4;92(9):e00815-26. doi: 10.1128/aem.00815-26 (PMC13607003; doi:10.1128/aem.00815-26)
Supplement: Supplementary file 1 [file aem.00815-26-s0001.docx]

**Supplementary Figures**

**Figure. A12.** Total ion current (TIC) chromatograms of AS19 and AS19-1. (A) Positive ion chromatogram of AS19; (B) Negative ion chromatogram of AS19; (C) Positive ion chromatogram of AS19-1; (D) Negative ion chromatogram of AS19-1.
