# Supplementary material for: Data-driven optimization of siderophore fermentation by Bacillus altitudinis AS19 and investigation of AS19’s antifungal properties against Candida albicans
Source: Appl Environ Microbiol. 2026 Aug 4;92(9):e00815-26. doi: 10.1128/aem.00815-26 (PMC13607003; doi:10.1128/aem.00815-26)
Supplement: Supplementary file 2 [file aem.00815-26-s0002.docx]

**Fig. A1.** (A) COG categories; (B) GO classification; (C) KEGG classification.

**Fig. A2.** The 11 biosynthetic gene clusters identified through antiSMASH secondary metabolite analysis. (A) other class; (B) terpene‑precursor class; (C) RiPP-like class; (D) terpene‑precursor class; (E) T3PKS class; (F) terpene class; (G) betalactone class; (H) betalactone class; (I) NRPS class; (J) RRE‑containing class; (K) terpene and NI‑siderophore class.

**Fig. A3.** Pareto chart.

**Fig. A4.** Three-dimensional conformations of the 20 AS19 siderophore analogs identified through virtual screening. (A) Woodybactin A; (B) Woodybactin B; (C) Woodybactin D; (D) Schizokinen; (E) Synechobactin A; (F) Synechobactin B; (G) Synechobactin C; (H) Fradimine B; (I) Nocardamine; (J) Nocardamin Glucuronide; (K) Clavamate B; (L) Ferrioxamine E; (M) Deferoxamine; (N) Desferrioxamine D1; (O) Desferrioxamine G; (P) Tenacibactin D; (Q) Bisucaberin B; (R) Legonoxamine A; (S) Legonoxamine B; (T) Carotenoid.

**Fig. A5.** Visualization of the optimal docking conformations of 24 representative complexes using PyMOL, and two‑dimensional protein–ligand interactions generated with Discovery Studio. (A) 2QZX-Woodybactin A; (B) 5TZ1-Woodybactin A; (C) FTR1-Woodybactin A; (D) SIT1-Woodybactin A; (E) 2QZX-Ferrioxamine E; (F) 5TZ1-Ferrioxamine E; (G) 5TZ1-Legonoxamine A; (H) SIT1-Ferrioxamine E; (I) 2QZX-Legonoxamine A; (J) 5TZ1-Legonoxamine A; (K) FTR1-Legonoxamine; (L) SIT1-Legonoxamine A; (M) 2QZX-Clavamate B; (N) 5TZ1-Clavamate B; (O) FTR1-Clavamate B; (P) SIT1-Clavamate B; (Q) 2QZX-Nocardamin Glucuronide; (R) 5TZ1-Nocardamin Glucuronide; (S) FTR1-Nocardamin Glucuronide; (T) SIT1-Nocardamin Glucuronide; (U) 2QZX-Nocardamine; (V) 5TZ1-Nocardamine; (W) FTR1-Nocardamine; (X) SIT1-Nocardamine.

**Fig. A6.** Root Mean Square Deviation (RMSD) Analysis of the Four Molecular Dynamics Simulation Systems. (A) Sap2-Nocardamin Glucuronide; (B) Sap2-Nocardamine; (C) CYP51-Nocardamin Glucuronide; (D) CYP51-Nocardamin Glucuronide.

**Fig. A7.** Radius of Gyration (Rg) and Solvent-Accessible Surface Area (SASA) of the Four MD Simulation Systems. (A-B) Sap2-Nocardamin Glucuronide; (C-D) Sap2-Nocardamine; (E-F) CYP51-Nocardamin Glucuronide; (G-H) CYP51-Nocardamin Glucuronide.

**Fig. A8.** Number of Hydrogen Bonds in the Four MD Simulation Systems. (A) Sap2-Nocardamin Glucuronide; (B) Sap2-Nocardamine; (C) CYP51-Nocardamin Glucuronide; (D) CYP51-Nocardamin Glucuronide.

**Fig. A9.** Root Mean Square Fluctuation (RMSF) of the Four MD Simulation Systems. (A) Sap2-Nocardamin Glucuronide; (B) Sap2-Nocardamine; (C) CYP51-Nocardamin Glucuronide; (D) CYP51-Nocardamin Glucuronide.

**Fig. A10.** Differential Metabolites Venn Diagram. AS19: The fermentation supernatant of Bacillus altitudinis AS19; AS19-1: AS19-1 (70 mg/mL).

**Fig. A11.** Extracted Ion Chromatogram (XIC) of Deferoxamine. Sample AS19: T2689936a; Sample AS19-1: T2689937a.

**Table A1** MIBiG Comparison of Terpene and NI-Siderophore Clusters

| Reference | **Similarity score** | **Type** | **Compound(s)** | **Organism** |
| --- | --- | --- | --- | --- |
| [BGC0002633.2](https://mibig.secondarymetabolites.org/go/BGC0002633.2) | 0.60 | other | schizokinen | Leptolyngbya sp. PCC 7376 |
| [BGC0002469.2](https://mibig.secondarymetabolites.org/go/BGC0002469.2) | 0.55 | other | woodybactin A, woodybactin B | Shewanella woodyi ATCC 51908 |
| [BGC0002683.2](https://mibig.secondarymetabolites.org/go/BGC0002683.2) | 0.55 | NRPS | schizokinen | Nostoc sp. PCC 7120 = FACHB-418 |
| [BGC0000645.3](https://mibig.secondarymetabolites.org/go/BGC0000645.3) | 0.50 | terpene | carotenoid | Halobacillus halophilus DSM 2266 |
| [BGC0001572.3](https://mibig.secondarymetabolites.org/go/BGC0001572.3) | 0.49 | other | desferrioxamine E | Pantoea agglomerans |
| [BGC0001478.5](https://mibig.secondarymetabolites.org/go/BGC0001478.5) | 0.48 | other | desferrioxamine E | Streptomyces sp. ID38640 |
| [BGC0000647.3](https://mibig.secondarymetabolites.org/go/BGC0000647.3) | 0.47 | terpene | carotenoid | Rhodobacter sphaeroides |
| [BGC0002692.2](https://mibig.secondarymetabolites.org/go/BGC0002692.2) | 0.46 | other | speibonoxamine, desoxy-desferrioxamine D1 | Kribbella speibonae |
| [BGC0000941.3](https://mibig.secondarymetabolites.org/go/BGC0000941.3) | 0.46 | other | desferrioxamin B | Streptomyces griseus subsp. griseus NBRC 13350 |
| [BGC0002305.2](https://mibig.secondarymetabolites.org/go/BGC0002305.2) | 0.45 | other | legonoxamine A, desferrioxamine B, legonoxamine B | Streptomyces sp. |

**Table** **A2** Factors and levels in the Plackett-Burman design

| Run on | Variable | Level | |
| --- | --- | --- | --- |
|  |  | -1 | 1 |
| A | pH | 6 | 7 |
| B | Temperature (°C) | 27 | 31 |
| C | Agitation Speed (r/min) | 170 | 190 |
| D | Sucrose (g/L) | 25 | 35 |
| E | Asparagine (g/L) | 2 | 6 |
| F | ZnSO_4_ (g/L) | 0.1 | 0.2 |
| G | CaCl_2_ (g/L) | 0.1 | 0.2 |
| H | MgSO_4_ (g/L) | 0.2 | 0.3 |
| J | NaCl (g/L) | 0.15 | 0.2 |
| K | K_2_HPO_4_ (g/L) | 0.1 | 0.2 |
| L | KH_2_PO_4_ (g/L) | 0.1 | 0.2 |

**Table A3** Analysis of Variance (ANOVA) Table for the Plackett-Burman Design

| Source | Sum of Squares | df | Mean Square | F-value | p-value |  |
| --- | --- | --- | --- | --- | --- | --- |
| Model | 524.57 | 7 | 74.94 | 17.4 | 0.0075 | significant |
| A-pH | 52.38 | 1 | 52.38 | 12.16 | 0.0252 |  |
| B- Temperature | 21.52 | 1 | 21.52 | 5 | 0.0891 |  |
| C- Agitation Speed | 63.16 | 1 | 63.16 | 14.67 | 0.0186 |  |
| D- Sucrose | 88.29 | 1 | 88.29 | 20.5 | 0.0106 |  |
| F- ZnSO_4_ | 45.12 | 1 | 45.12 | 10.48 | 0.0318 |  |
| H- MgSO_4_ | 17.98 | 1 | 17.98 | 4.18 | 0.1105 |  |
| L- KH_2_PO_4_ | 236.12 | 1 | 236.12 | 54.83 | 0.0018 |  |
| Residual | 17.22 | 4 | 4.31 |  |  |  |
| Cor Total | 541.8 | 11 |  |  |  |  |
| R² | 0.9682 | Adeq Precision | 14.1441 |  |  |  |
| Adjusted R² | 0.9126 | C.V. % | 4.49 |  |  |  |

**Table** **A4** Factors and Levels in the Box-Behnken Design

| Run on | Variable | Level | | |
| --- | --- | --- | --- | --- |
|  |  | -1 | 0 | 1 |
| A | pH | 6 | 6.5 | 7 |
| B | Sucrose (g/L) | 27 | 29 | 31 |
| C | KH_2_PO_4_ (g/L) | 170 | 180 | 190 |

**Table** **A5** Analysis of Variance (ANOVA) Table for the Box-Behnken Design

| Source | Sum of Squares | df | Mean Square | F-value | p-value |  |
| --- | --- | --- | --- | --- | --- | --- |
| Model | 107.73 | 9 | 11.97 | 61.66 | < 0.0001 | significant |
| A-pH | 2.42 | 1 | 2.42 | 12.47 | 0.0096 |  |
| B- Sucrose | 1.1 | 1 | 1.1 | 5.64 | 0.0492 |  |
| C- KH_2_PO_4_ | 0.0684 | 1 | 0.0684 | 0.3526 | 0.5713 |  |
| AB | 0.0121 | 1 | 0.0121 | 0.0623 | 0.81 |  |
| AC | 1.21 | 1 | 1.21 | 6.23 | 0.0412 |  |
| BC | 0.0529 | 1 | 0.0529 | 0.2725 | 0.6178 |  |
| A² | 41.63 | 1 | 41.63 | 214.45 | < 0.0001 |  |
| B² | 19.91 | 1 | 19.91 | 102.55 | < 0.0001 |  |
| C² | 30.8 | 1 | 30.8 | 158.63 | < 0.0001 |  |
| Residual | 1.36 | 7 | 0.1941 |  |  |  |
| Lack of Fit | 0.71 | 3 | 0.2367 | 1.46 | 0.3518 | not significant |
| Pure Error | 0.6489 | 4 | 0.1622 |  |  |  |
| Cor Total | 109.09 | 16 |  |  |  |  |
| R² | 0.9875 |  | Adeq Precision | 20.8369 |  |  |
| Adjusted R² | 0.9715 |  | C.V. % | 0.7005 |  |  |

**Table A6** Molecular Docking Results

| Chemical compound | Protein(kcal/mol) | | | |
| --- | --- | --- | --- | --- |
|  | 2QZX | 5TZ1 | FTR1 | SIT1 |
| Woodybactin A | -6.9 | -6.6 | -6.6 | -8.4 |
| Bisucaberin B | -6.6 | -6.4 | -6.2 | -6.4 |
| FerrioxamineE | 8.7 | -8.8 | -7.6 | -7.8 |
| Legonoxamine A | -7.2 | -8.3 | -7.2 | -8.3 |
| Woodybactin B | -7.0 | -7.0 | -6.2 | -7.6 |
| Woodybactin D | -6.5 | -6.5 | -6.1 | -7.4 |
| schizokinen | -6.3 | -6.3 | -5.4 | -6.8 |
| Synechobactin A | -6.4 | -6.3 | -5.2 | -7.5 |
| Synechobactin B | -5.3 | -6.5 | -5.9 | -6.8 |
| Synechobactin C | -6.0 | -7.1 | -6.0 | -6.5 |
| Clavamate B | -11.1 | -10.7 | -9.9 | -9.0 |
| Nocardamin Glucuronide | -10.0 | -9.0 | -8.8 | -8.1 |
| Nocardamine | -9.3 | -9.3 | -8.6 | -7.8 |
| carotenoid | -5.6 | -6.0 | -5.3 | -5.5 |
| Deferoxamine | -5.6 | -6.0 | -5.8 | -5.7 |
| Desferrioxamine D1 | -5.8 | -7.0 | -6.4 | -4.5 |
| Desferrioxamine G | -5.6 | -7.0 | -5.7 | -4.8 |
| Fradimine B | -6.8 | -7.3 | -5.7 | -7.6 |
| Legonoxamine B | -6.0 | -6.7 | -5.1 | -5.0 |
| Tenacibactin D | -4.8 | -7.0 | -6.1 | -5.2 |

Note: A binding energy lower than -5.0 kcal/mol is considered indicative of potential binding activity, while values below -7.0 kcal/mol are prioritized as candidate compounds.

**Table A7** The results show the information of the minimum binding energy, the number of hydrogen bonds and the specific binding sites of the 20 candidate iron carriers screened with 2QZX, 5TZ1, FTR1 and SIT1.

| Component | Minimum Binding energy (Kcal/mol) | Nnmber of hydrogen bonds | Amino acid residus |
| --- | --- | --- | --- |
| 2QZX-Woodybactin A | -6.9 | 8 | TYR-225, ILE-223, THR-222, GLY-220, GLY-85, ASP-86, ASP-32 |
| 2QZX-Bisucaberin B | -6.6 | 14 | TYR-225, THP-222, THP-221, ASP-218, GLY-220, ASP-86, SER-88, ARG-120, GLY-220, ASP-32 |
| 2QZX-Ferrioxamine E | -8.7 | 3 | ARG-297, ARG-299, TYR-225 |
| 2QZX-Legonoxamine A | -7.2 | 10 | SER-88, ARG-120, SER-61, TRP-51, GLY-220, THR-222, ARG-29 |
| 2QZX-Woodybactin B | -7.0 | 8 | TYR-225, THR-222, GLY-220, ASP-32, ASP-86 |
| 2QZX-Woodybactin D | -6.5 | 9 | ARG-299, TYR-225, ILE-223, THR-222 |
| 2QZX-schizokinen | -6.3 | 6 | ASP-218, ASP-32, THR-221, GLY-220, ASP-32, GLY-85 |
| 2QZX-Synechobactin A | -6.4 | 3 | ASP-86, GLY-220 |
| 2QZX-Synechobactin B | -5.3 | 5 | GLY-85, THR-221, GLY-220, GLY-34, ASP-32 |
| 2QZX-Synechobactin C | -6.0 | 9 | GLY-85, TYR-225, GLY-220, ASP-21, THR-221, THR-22 |
| 2QZX-Clavamate B | -11.1 | 3 | TYR-225, GLU-300, SER-301 |
| 2QZX-Nocardamin Glucuronide | -10.0 | 7 | GLY-220, THR-221, THR-222, TYR-225, GLU-300, ALA-147, ARG-52 |
| 2QZX-Nocardamine | -9.3 | 5 | TYR-225, ARG-299, ASP-86, GLY-220, ASP-218 |
| 2QZX-carotenoid | -5.6 | 1 | THR-222 |
| 2QZX-Deferoxamine | -5.6 | 5 | ASP-32, GLY-34, GLY-220, ASP-218, ASP-303 |
| 2QZX-Desferrioxamine D1 | -5.8 | 6 | SER-88, ASP-86, THR-221, ARG-297, ARG-299 |
| 2QZX-Desferrioxamine G | -5.6 | 6 | SER-88, GLY-220, ASP-86, SER-301, ASP-303, TYR-225 |
| 2QZX-Fradimine B | -6.8 | 9 | THR-13, ILE-12, ASP-32, GLY-220, THR-221, THR-222, TYR-225, GLY-85 |
| 2QZX-Legonoxamine B | -6.0 | 6 | ASP-32, GLY-34, GLY-220, ASP-218, THR-221, THR-222 |
| 2QZX-Tenacibactin D | -4.8 | 7 | LEU-280, SER-277, GLU-278, GLN-282, ALA-162, ASN-9 |
| 5TZ1-Woodybactin A | -6.6 | 3 | ARG-381, HIS-468, TYR-132 |
| 5TZ1-Bisucaberin B | -6.4 | 7 | TYR-132, TYR-118, ARG-381, HIS-468, PRO-462 |
| 5TZ1-FerrioxamineE | -8.8 | 3 | ASP-502, THR-411, GLU-73 |
| 5TZ1-Legonoxamine A | -8.3 | 6 | TYR-505, MET-508, GLY-303, TYR-132, LYS-143 |
| 5TZ1-Woodybactin B | -7.0 | 7 | ARG-381, TYR-132, LYS-143, GLY-303 |
| 5TZ1-Woodybactin D | -6.5 | 4 | TYP-132, HIS-468, PHE-463, LYS-143 |
| 5TZ1-schizokinen | -6.3 | 3 | TYR-132, HIS-468 |
| 5TZ1-Synechobactin A | -6.3 | 8 | TYR-132, ARG-381, HIS-468, ARG-469, PHE-463, ILE-471 |
| 5TZ1-Synechobactin B | -6.5 | 3 | TYR-132, HIS-468, ILE-471 |
| 5TZ1-Synechobactin C | -7.1 | 6 | TYR-132, HIS-468, LYS-143, PHE-463 |
| 5TZ1-Clavamate B | -10.7 | 2 | SER-222, GLU-194 |
| 5TZ1-Nocardamin Glucuronide | -9.0 | 6 | GLN-77,SER-74, THR-411, GLU-420, MET-372, TYR-505 |
| 5TZ1-Nocardamine | -9.3 | 4 | GLU-420, MET-372, TYR-505 |
| 5TZ1-carotenoid | -6.0 | 5 | SER-438, GLY-455, ARG-469 |
| 5TZ1-Deferoxamine | -6.0 | 6 | LYS-190, GLU-194, ASP-173, THP-172, GLU-165 |
| 5TZ1-Desferrioxamine D1 | -7.0 | 5 | ARG-381, MET-508, THR-311, GLY-303 |
| 5TZ1-Desferrioxamine G | -7.0 | 9 | TYR-505, PRO-230, GLY-303, TYP-132, TYR-118, ARG-381, SER-378 |
| 5TZ1-Fradimine B | -7.3 | 9 | MET-508, TYR-118, SER, -378, HIS-377 |
| 5TZ1-Legonoxamine B | -6.7 | 3 | SER-378, HIS-377 |
| 5TZ1-Tenacibactin D | -7.0 | 7 | SER-378, HIS-468, PHE-463, TYR-132 |
| FTR1-Woodybactin A | -6.6 | 13 | ASN-172, GLU-89, ARG-226, ASN-178, ASN-275, GLN-284, ASP-86, TYR-230, GLU-269, SER-245 |
| FTR1-Bisucaberin B | -6.2 | 4 | SER-128, GLN-358, ASN-359, PHE-360 |
| FTR1-FerrioxamineE | -7.6 | 4 | GLU-342, LYS-336, ARG-109 |
| FTR1-Legonoxamine A | -7.2 | 14 | LYS-78, ASP-3, ASN-278, ARG-226, TYR-230, GLU-89, ASP-86, GLU-85, LEU-171, ASN-172 |
| FTR1-Woodybactin B | -6.2 | 9 | PHE-5, VAL-7, ASN-6, ASP-275, SER-173, ASN-278, ARG-226 |
| FTR1-Woodybactin D | -6.1 | 12 | PHE-5, ASP-275, ASN-278, ARG-226, TYR-230, PRO-268, GLU-269, |
| FTR1-schizokinen | -5.4 | 4 | LEU-281, GLN-284, SER-291 |
| FTR1-Synechobactin A | -5.2 | 2 | TRP-52, TYR-198 |
| FTR1-Synechobactin B | -5.9 | 7 | LYS-143, LYS-144, GLN-50, LEU-125, TYR-140 |
| FTR1-Synechobactin C | -6.0 | 6 | TYR-197, SER-25, LYS-32, TYP-52 |
| FTR1-Clavamate B | -9.9 | 3 | GLU-312, LYS-340, GLY-337 |
| FTR1-Nocardamin Glucuronide | -8.8 | 4 | TYR-198, TRP-52, GLY-56, LYS-45 |
| FTR1-Nocardamine | -8.6 | 1 | MET-332 |
| FTR1-carotenoid | -5.3 | 0 | - |
| FTR1-Deferoxamine | -5.8 | 4 | ASN-83, SER-245, LEU-171, SEP-173 |
| FTR1-Desferrioxamine D1 | -6.4 | 4 | GLU-368, ARG-135, LYS-143 |
| FTR1-Desferrioxamine G | -5.7 | 1 | ASN-131 |
| FTR1-Fradimine B | -5.7 | 3 | TYR-295, LEU-281, SER-291 |
| FTR1-Legonoxamine B | -5.1 | 2 | LEU-281, SER-225 |
| FTR1-Tenacibactin D | -6.1 | 4 | GLN-358, LYS-143, GLN-50 |
| SIT1-Woodybactin A | -8.4 | 8 | THR-573, LEU-180, LEU-578, SER-463, GLN-462, VAL-459 |
| SIT1-Bisucaberin B | -6.4 | 7 | GLU-575, LEU-180, THR-573, ARG-181 |
| SIT1-FerrioxamineE | -7.8 | 3 | GLN-377, SER-378, ALA-206 |
| SIT1-Legonoxamine A | -8.3 | 12 | SER-388, SER-391, TYR-455, ASN-102, TYR-485, THR-105, ARG-482, GLN-160 |
| SIT1-Woodybactin B | -7.6 | 8 | THR-573, LEU-578, GLN-462, VAN-459 |
| SIT1-Woodybactin D | -7.4 | 12 | GLN-79, SER-388, GLN-160, THR-105, ARG-482, TYR-481, SER-385 |
| SIT1-schizokinen | -6.8 | 12 | ALA-384, SER-78, GLN-160, THR-105, TYR-485, ASP-355, ARG-482, TYR-481 |
| SIT1-Synechobactin A | -7.5 | 4 | SER-388, TYR-163, TYR-485, ARG-482 |
| SIT1-Synechobactin B | -6.8 | 6 | GLN-160, TYR-163, SER-78, SER-388, TYR-485 |
| SIT1-Synechobactin C | -6.5 | 8 | SER-388, SER-391, SER-78, TYR-455, TYR-481, TYR-163, TYR-485 |
| SIT1-Clavamate B | -9.0 | 0 | - |
| SIT1-Nocardamin Glucuronide | -8.1 | 5 | ARG-183, HIS-467, GLU-468 |
| SIT1-Nocardamine | -7.8 | 4 | GLU-94, SER-96, LEU-294, ASN-150 |
| SIT1-carotenoid | -5.5 | 2 | LYS-60, HIS-55 |
| SIT1-Deferoxamine | -5.7 | 3 | ALA-384, SER-388, SER-474 |
| SIT1-Desferrioxamine D1 | -4.5 | 2 | TYR-404, ARG-407 |
| SIT1-Desferrioxamine G | -4.8 | 6 | ASP-580, ARG-183, SER-175, GLU-468, SER-40 |
| SIT1-Fradimine B | -7.6 | 8 | VAL-101, ASN-102, THR-105, ARG-482, GLN-160, ALA-384, SER-78 |
| SIT1-Legonoxamine B | -5.0 | 2 | LEU-294 |
| SIT1-Tenacibactin D | -5.2 | 6 | ARG-181, ASN-574, LEU-180 |

**Table A8** Differential Metabolites of Deferoxamine

| Compounds | Formula | AS19-1 | AS19 | Log2FC | Type |
| --- | --- | --- | --- | --- | --- |
| Deferoxamine | C25H48N6O8 | 26835835.16 | 1221550.27 | 4.4574 | up |

Note: AS19: The fermentation supernatant of Bacillus altitudinis AS19; AS19-1: AS19-1 (70 mg/mL)

**Table A9** Antimicrobial activity of AS19 cell‑free supernatant in vitro

| Species |  | 无细胞上清 | |
| --- | --- | --- | --- |
|  | LB | | 缺铁 |
| Candida albicans | - | | ++ |
| Porphyromonas gingivalis | - | | - |
| Fusobacterium nucleatum | - | | - |
| Escherichia coli | - | | - |
| Staphylococcus aureus | - | | - |
| Acinetobacter baumannii | - | | - |
| Pseudomonas aeruginosa | - | | - |
| Klebsiella pneumoniae | - | | - |

Note: –, no antimicrobial activity; +, antimicrobial activity (inhibition zone < 10 mm); ++, significant antimicrobial activity (inhibition zone ≥ 10 mm).
